# A new approach to digitized cognitive monitoring: validity of the SelfCog in Huntington’s disease

**DOI:** 10.1093/braincomms/fcad043

**Published:** 2023-03-06

**Authors:** Marine Lunven, Karen Hernandez Dominguez, Katia Youssov, Jennifer Hamet Bagnou, Rafika Fliss, Henri Vandendriessche, Blanche Bapst, Graça Morgado, Philippe Remy, Robin Schubert, Ralf Reilmann, Monica Busse, David Craufurd, Renaud Massart, Anne Rosser, Anne-Catherine Bachoud-Lévi

**Affiliations:** Département d'Etudes Cognitives, École normale supérieure, PSL University, 75005 Paris, France; University Paris Est Creteil, INSERM U955, Institut Mondor de Recherche Biomédicale, Equipe NeuroPsychologie Interventionnelle, F-94010 Creteil, France; AP-HP, Hôpital Henri Mondor-Albert Chenevier, Centre de référence Maladie de Huntington, Service de Neurologie, F-94010 Créteil, France; NeurATRIS, Hôpital Henri Mondor, 94010 Créteil, France; Département d'Etudes Cognitives, École normale supérieure, PSL University, 75005 Paris, France; University Paris Est Creteil, INSERM U955, Institut Mondor de Recherche Biomédicale, Equipe NeuroPsychologie Interventionnelle, F-94010 Creteil, France; AP-HP, Hôpital Henri Mondor-Albert Chenevier, Centre de référence Maladie de Huntington, Service de Neurologie, F-94010 Créteil, France; NeurATRIS, Hôpital Henri Mondor, 94010 Créteil, France; Département d'Etudes Cognitives, École normale supérieure, PSL University, 75005 Paris, France; University Paris Est Creteil, INSERM U955, Institut Mondor de Recherche Biomédicale, Equipe NeuroPsychologie Interventionnelle, F-94010 Creteil, France; AP-HP, Hôpital Henri Mondor-Albert Chenevier, Centre de référence Maladie de Huntington, Service de Neurologie, F-94010 Créteil, France; NeurATRIS, Hôpital Henri Mondor, 94010 Créteil, France; Département d'Etudes Cognitives, École normale supérieure, PSL University, 75005 Paris, France; University Paris Est Creteil, INSERM U955, Institut Mondor de Recherche Biomédicale, Equipe NeuroPsychologie Interventionnelle, F-94010 Creteil, France; AP-HP, Hôpital Henri Mondor-Albert Chenevier, Centre de référence Maladie de Huntington, Service de Neurologie, F-94010 Créteil, France; NeurATRIS, Hôpital Henri Mondor, 94010 Créteil, France; Département d'Etudes Cognitives, École normale supérieure, PSL University, 75005 Paris, France; University Paris Est Creteil, INSERM U955, Institut Mondor de Recherche Biomédicale, Equipe NeuroPsychologie Interventionnelle, F-94010 Creteil, France; AP-HP, Hôpital Henri Mondor-Albert Chenevier, Centre de référence Maladie de Huntington, Service de Neurologie, F-94010 Créteil, France; NeurATRIS, Hôpital Henri Mondor, 94010 Créteil, France; Département d'Etudes Cognitives, École normale supérieure, PSL University, 75005 Paris, France; University Paris Est Creteil, INSERM U955, Institut Mondor de Recherche Biomédicale, Equipe NeuroPsychologie Interventionnelle, F-94010 Creteil, France; AP-HP, Hôpital Henri Mondor-Albert Chenevier, Centre de référence Maladie de Huntington, Service de Neurologie, F-94010 Créteil, France; NeurATRIS, Hôpital Henri Mondor, 94010 Créteil, France; Department of Neuroradiology, AP-HP, Henri Mondor University Hospital, 94010 Créteil, France; Faculty of Medicine, Université Paris Est Créteil, F-94010 Créteil, France; Inserm, Centre d’Investigation Clinique 1430, APHP, Hôpital Henri Mondor, 94010 Créteil, France; Département d'Etudes Cognitives, École normale supérieure, PSL University, 75005 Paris, France; University Paris Est Creteil, INSERM U955, Institut Mondor de Recherche Biomédicale, Equipe NeuroPsychologie Interventionnelle, F-94010 Creteil, France; AP-HP, Hôpital Henri Mondor-Albert Chenevier, Centre de référence Maladie de Huntington, Service de Neurologie, F-94010 Créteil, France; NeurATRIS, Hôpital Henri Mondor, 94010 Créteil, France; George Huntington Institute, Technology-Park, 48149 Muenster, Germany; Department of Neurodegeneration and Hertie Institute for Clinical Brain Research, University of Tuebingen, 72076 Tuebingen, Germany; George Huntington Institute, Technology-Park, 48149 Muenster, Germany; Department of Neurodegeneration and Hertie Institute for Clinical Brain Research, University of Tuebingen, 72076 Tuebingen, Germany; Department of Clinical Radiology, University of Muenster, 48149 Muenster, Germany; Centre for Trials Research, Cardiff University, Cardiff CF14 4EP, UK; Wales Brain Research And Intracranial Neurotherapeutics (BRAIN) Biomedical Research Unit, College of Biomedical and Life Sciences, Cardiff University, Cardiff CF14 4EP, UK; Manchester Centre for Genomic Medicine, St Mary’s Hospital, Manchester University NHS Foundation Trust, Manchester Academic Health Science Centre, Manchester M13 9PL, UK; Division of Evolution and Genomic Sciences, School of Biological Sciences, Faculty of Biology, Medicine and Health, University of Manchester, Manchester Academic Health Science Centre, Manchester M13 9PL, UK; Département d'Etudes Cognitives, École normale supérieure, PSL University, 75005 Paris, France; University Paris Est Creteil, INSERM U955, Institut Mondor de Recherche Biomédicale, Equipe NeuroPsychologie Interventionnelle, F-94010 Creteil, France; AP-HP, Hôpital Henri Mondor-Albert Chenevier, Centre de référence Maladie de Huntington, Service de Neurologie, F-94010 Créteil, France; NeurATRIS, Hôpital Henri Mondor, 94010 Créteil, France; Wales Brain Research And Intracranial Neurotherapeutics (BRAIN) Biomedical Research Unit, College of Biomedical and Life Sciences, Cardiff University, Cardiff CF14 4EP, UK; Cardiff School of Medicine, Neuroscience and Mental Health Institute, Cardiff CF24 4HQ, UK; School of Biosciences, Cardiff University Brain Repair Group, Cardiff CF10 3AX, UK; Département d'Etudes Cognitives, École normale supérieure, PSL University, 75005 Paris, France; University Paris Est Creteil, INSERM U955, Institut Mondor de Recherche Biomédicale, Equipe NeuroPsychologie Interventionnelle, F-94010 Creteil, France; AP-HP, Hôpital Henri Mondor-Albert Chenevier, Centre de référence Maladie de Huntington, Service de Neurologie, F-94010 Créteil, France; NeurATRIS, Hôpital Henri Mondor, 94010 Créteil, France

**Keywords:** neurodegenerative disease, Huntington’s disease, digitized cognitive assessment, longitudinal follow-up, clinical trial

## Abstract

Cognitive deficits represent a hallmark of neurodegenerative diseases, but evaluating their progression is complex. Most current evaluations involve lengthy paper-and-pencil tasks which are subject to learning effects dependent on the mode of response (motor or verbal), the countries’ language or the examiners. To address these limitations, we hypothesized that applying neuroscience principles may offer a fruitful alternative. We thus developed the SelfCog, a digitized battery that tests motor, executive, visuospatial, language and memory functions in 15 min. All cognitive functions are tested according to the same paradigm, and a randomization algorithm provides a new test at each assessment with a constant level of difficulty.

Here, we assessed its validity, reliability and sensitivity to detect decline in early-stage Huntington’s disease in a prospective and international multilingual study (France, the UK and Germany). Fifty-one out of 85 participants with Huntington’s disease and 40 of 52 healthy controls included at baseline were followed up for 1 year. Assessments included a comprehensive clinical assessment battery including currently standard cognitive assessments alongside the SelfCog. We estimated associations between each of the clinical assessments and SelfCog using Spearman’s correlation and proneness to retest effects and sensitivity to decline through linear mixed models. Longitudinal effect sizes were estimated for each cognitive score. Voxel-based morphometry and tract-based spatial statistics analyses were conducted to assess the consistency between performance on the SelfCog and MRI 3D-T1 and diffusion-weighted imaging in a subgroup that underwent MRI at baseline and after 12 months.

The SelfCog detected the decline of patients with Huntington’s disease in a 1-year follow-up period with satisfactory psychometric properties. Huntington’s disease patients are correctly differentiated from controls. The SelfCog showed larger effect sizes than the classical cognitive assessments. Its scores were associated with grey and white matter damage at baseline and over 1 year. Given its good performance in longitudinal analyses of the Huntington’s disease cohort, it should likely become a very useful tool for measuring cognition in Huntington’s disease in the future. It highlights the value of moving the field along the neuroscience principles and eventually applying them to the evaluation of all neurodegenerative diseases.

## Teaser

Lunven *et al*. developed a brief digitized cognitive battery, assessing cognitive functions following a similar procedure in Huntington’s disease. Cognitive decline over 1 year with greater sensitivity than traditional tests was observed in a multicentric study. It will likely improve patients’ follow-up by detecting eventual heterogeneous progression of cognitive performance.

## Graphical Abstract

**Graphical abstract:**
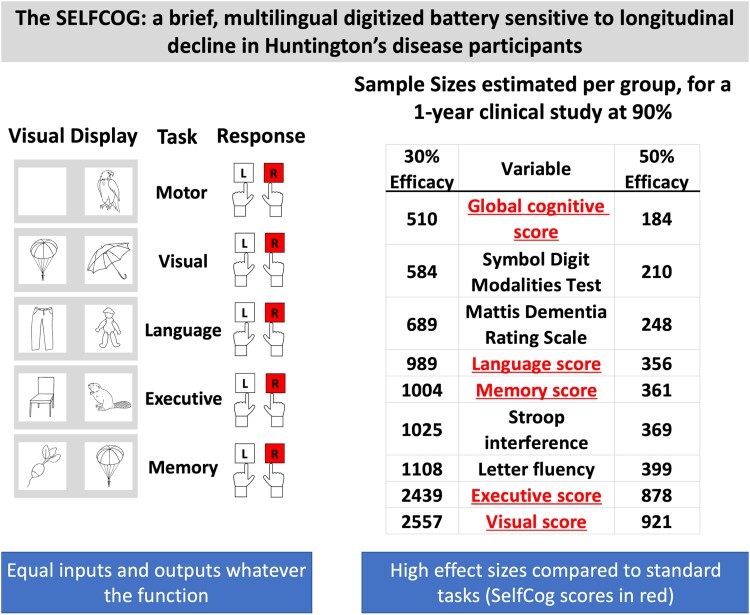

## Introduction

Assessment of cognitive capacities is a cornerstone in the clinical evaluation of neurodegenerative disorders (NDDs).^1^ As the genetic or neurobiological mechanisms underlying specific forms of NDD are discovered, promising targeted treatments to stop or modify the natural course of the diseases are on the horizon.^2^ Despite these exciting developments and the progress made to statistically model disease progression^3^ or to generate multidimensional global score,^4^ the need to improve cognitive assessment to match these advances remains.^5^ Indeed, current common approaches to cognitive assessment use a set of multi-item rating scales and batteries of brief cognitive tasks. Most of them are paper-and-pencil based, language and education-dependent, time-consuming and expensive as they must be administered in person by a healthcare professional. The cognitive scores are affected by floor, ceiling and learning effects and are prone to inter-rater and intra-rater variability.^3,6^ Other limitations of classical paper-and-pencil tasks stem from the fact that most often cognitive processing cannot be disentangled from perceptual and motor processing [as in the Symbol Digit Modalities Test (SDMT)],^7^ which can be a major limitation in individuals with motor impairment. Differentiating between cognitive impairments can also be difficult with most tasks, for example, distinguishing between language and executive impairments using verbal fluency.^8^ The lack of specificity in cognitive assessment is particularly problematic since impairments often vary from patient to patient and interventions may have a selective impact on certain functions, for example, when using molecules targeting specific receptors.^9^ In addition, comparing changes in cognitive functions is difficult regarding the variable number of parallel forms across different tests [e.g. six forms for the Hopkins Verbal Learning Test (HVLT)^10^ versus one for the Mini-Mental State Examination]. Most classical tasks do not offer equivalent parallel forms,^11,12^ which leads to a retest effect due to repetition during serial assessments and therefore to underestimation of cognitive decline.^13,14^ Finally, the development of a single composite score, reflecting global cognitive abilities, from currently used clinical assessments, has as yet not been achieved despite numerous attempts.^15^

Digitized cognitive tasks are emerging as one potential solution to overcome paper-and-pencil task limitations. They allow greater sensitivity and finer analysis of cognitive processing by precisely recording accuracy and response time (RT) in milliseconds.^16^ Among them, the Cambridge Neuropsychological Test Automated Battery (CANTAB) is the cognitive battery the most widely used for neurological conditions in publications.^17,18^ However, CANTAB cognitive profiling requires lengthy assessments (> 30 min), the presence of an examiner to supervise the evaluation and the licence is expensive. The diversity of the procedures of its different components makes comparisons of performance difficult. Fatigue may then bias assessments in individuals with NDD. Sensitivity of tasks to cognitive impairments in NDD is demonstrated, and such of them are able to detect subtle deficit in pre-clinical populations such as in Huntington’s disease.^17^ However, the sensitivity to longitudinal cognitive change could be limited. For example, 17/19 tests from the CANTAB failed to record significant deterioration in Huntington’s disease participants over a decade of follow-up as part of a neural transplant trial.^19^ Despite its ability to distinguish subjects with cognitive impairments from healthy controls, most CANTAB tests are not specific enough to distinguish one cognitive function from another.^20^

To overcome these shortcomings, we hypothesize that applying the principles of experimental cognitive neurosciences to digitized assessments in clinics is required to move the field forward. For example, functional MRI design^21^ tends to equalize all parameters except the one of interest when comparing conditions. To avoid bias from stimuli presentation and order effect, all stimuli are randomized. We thus developed a brief (around 15 min) digitized cognitive battery, the SelfCog, setting up a constant paradigm to assess language, visual, executive and memory functions as well as motor performance. At each trial, after the presentation of two images, the participant is asked to press one of the two response keys. Only the instruction changes across the tasks. We recorded both the accuracy and the response time. Thus, the SelfCog makes it possible to isolate cognitive processing times from motor response times, which can greatly vary from one patient to another. To limit the retest effect^14^—the fact that performance improves at the second exposure when experiments are presented twice—new images are presented at each assessment while keeping the cognitive load constant. Considering the need for a single primary end-point in clinical studies, the SelfCog proposes a sub-score for each function and an overall combined global score balanced between the different functions. The SelfCog has to date been developed for application in French, German and English, and its use is accessible to non-experts with minimal training.^12^

In this study, we focused on Huntington’s disease to assess the initial validity and value of the SelfCog in clinical practice. It is an autosomal dominant NDD characterized by motor, cognitive and psychiatric impairments.^4,22,23^ Unlike most NDDs, disease onset can be estimated by genetic testing, facilitating the study of animal models for the development of experimental and clinical therapeutics.^24^ In this context, Huntington’s disease represents an excellent candidate and model of NDD to test the value of the SelfCog in evaluating the disease progression. We assessed the construct validity of the SelfCog with commonly used classical paper-and-pencil cognitive tasks and structural brain imaging. We showed its test–retest reliability and utility for micro-monitoring cognitive decline over 1 year in a subgroup of Huntington’s disease participants.

## Materials and methods

### Participants

Eighty-five Huntington’s disease patients and 52 healthy controls were included in a prospective multicentre international observational study as part of the European Repair-HD consortium (Table 1; https://cordis.europa.eu/project/id/602245) dedicated to the development of clinical assessments to test novel therapies. Participants were recruited from four sites: two focused on cross-sectional follow-up (M0) at Manchester, UK (*n* = 10), and Muenster, Germany (*n* = 34), and two extended evaluations to optional longitudinal follow-up at Cardiff, UK (*n* = 19), and Créteil, France (*n* = 74), by eventually adding Month 1 (M1) and Month 12 (M12) evaluations. This yielded the follow-up of 74 Huntington’s disease participants and 50 controls up to M1 (Supplementary Table 1) and 51 Huntington’s disease participants and 40 controls up to M12 (Supplementary Table 2). There was no significant difference in demographic data between participants included at M0 and the remaining participants evaluated at M12 (Supplementary Table 3).

**Table 1:** Demographics and neurological descriptions for controls and Huntington’s disease participants at M0 and M1

	Controls	Huntington’s disease participants	*P*-values of controls versus Huntington’s disease participants
Age	50.65 ± 10.20 (26.16–69.96)	52.06 ± 10.8 (23.19–78.06)	0.45
Sex	27F/25M	33F/52M	0.13
Education level	14.06 ± 2.83 (8.00–24.00)	13.98 ± 2.92 (9.00–20.00)	0.87
Laterality	2A/6L/44R	7L/78R	0.15
CAG	–	43.76 ± 3.69 (38.00–62.00)^a^	
Age at onset	–	47.49 ± 10.82 (20.02–73.83)^b^	
Disease duration	–	4.53 ± 3.81 (0.00–19.18)^b^	
DBS	–	400.03 ± 97.81 (129.62–672.50)^a^	
TFC	13.00 ± 0.00 (13.00–13.00)	10.52 ± 1.78 (7.00–13.00)	<2 e^−16^
TMS	0.56 ± 1.15 (0.00–5.00)	29.68 ± 14.58 (1.00–60.00)	<2 e^−16^

Unless otherwise specified, quantitative values are means ± standard deviations and range [ ]. F, female; M, male; A, ambidextrous; L, left; R, right; TFC, Total Functional Capacity; TMS, Total Motor Score; CAG, cytosine–adenine–guanine; DBS, disease burden score = Age × (CAG repeat—35.5). ^a^One missing data point. ^b^Five missing data points.

The inclusion criteria for Huntington’s disease were (i) confirmed cytosine–adenine–guanine (CAG) expansion (≥ 36 CAG repeats) and (ii) stages 1 or 2 of the disease according to the Unified Huntington’s Disease Rating Scale (UHDRS) Total Functional Capacity (TFC) scores (TFC ≥ 7).^25^ Matched healthy controls were spouses or partners of Huntington’s disease participants, gene-negative siblings or persons unrelated to the Huntington’s disease participants with a Mattis Dementia Rating Scale (MDRS) global score ≥ 136.^26^ Exclusion criteria for controls included alcohol or substance abuse and neurological co-morbidity. Participants’ demographics and pathological disease burden scores (DBS)^27^ are reported in Table 1. Written informed consent was obtained from each participant. The Core Assessment Protocol for Innovative Therapy-HD Beta study was approved by the ethical committee (NCT 03119246, https://clinicaltrials.gov/ct2/show/NCT03119246?cond=Huntington+Disease&cntry=FR&draw=2).

### General assessment

The neurological evaluation comprises the Total Motor Score (TMS) for which raters are certified annually^28^ and the TFC from the UHDRS.^8^ Participants performed also classical paper-and-pencil cognitive tasks with the Letter Verbal Fluency Task over 1 min,^29^ the Symbol Digit Modalities Test (SDMT), the Stroop test (colour, word and interference), the Mattis Dementia Rating Scale (MDRS),^26^ the categorical fluency task and the Hopkins Verbal Learning Memory Test (HVLMT).^10^ To minimize learning effects, alternative parallel forms were used in a fixed order for each assessment of the Hopkins Verbal Learning Test (HVLT).^11^ A subgroup of patients underwent MRI brain imaging.

### SelfCog assessment

**Figure 1:**
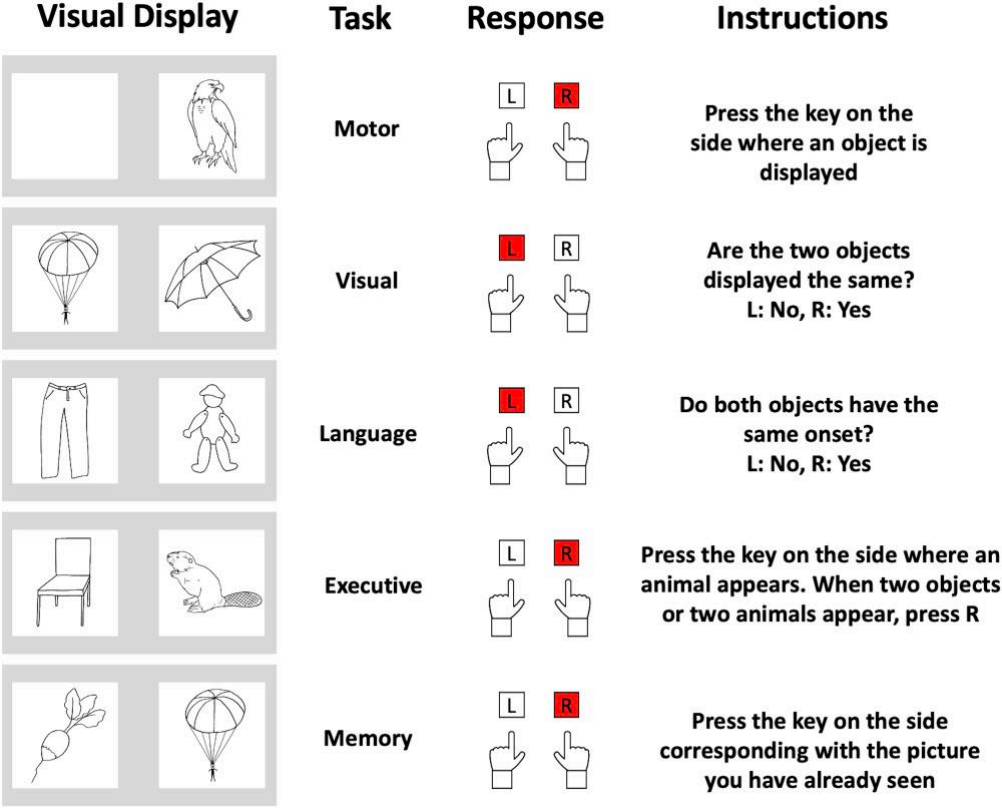
**Schematic of the SelfCog battery.** Each trial consists of two pictures. The participant is asked to press the key on a computer keyboard that corresponds to its answer. Instructions vary for each task. The order of the subset was kept fixed.

The SelfCog (IDDN FR.001.230010.000.S.A.2020.000.31230) consists in a digitized task divided in five subsequent subtests that explore motor, visuospatial, language, executive and memory functions (Fig. 1). Each subtest included a short training phase (including four trials) and then a test phase of 40 trials. The whole SelfCog totalled 200 test trials in around 15 min.

For each trial of each subtest, the input stimuli (two images presented on the screen) and output responses (pressing a key) are similar, the only variations being in the instructions. To make each assessment unique and to minimize learning effects related to multiple exposures while maintaining an equivalent level of difficulty, we built a randomization algorithm to select stimuli from a list of 585 possible pairs of black line pictures, designed according to the constraints of each subtest and language (French, English and German) (see Supplementary Appendix 1 for details). Images appeared on a computer screen in two white squares on a grey background and remained displayed until the participant either pressed a button according to the instructions or reached a timeout defined for each subtest. Participants were instructed to respond as fast as possible while trying to avoid errors. Stimulus presentation and response recording were performed in Python, using the PsychoPy toolbox (https://www.psychopy.org/).

### Assessment time-course

Participants were assessed at M0 and optionally at M1 and M12. However, as motor and functional assessments are not known to be sensitive to the retest effect,^14^ assessment at M1 was offered only for the cognitive assessment, while the full set of assessments was offered at M0 and M12 at sites with the capacity to perform them. The M1 assessment allows to compare psychometric properties and retest effect of the SelfCog and classical paper-and-pencil tests.^14^

### Statistical analysis

#### General assessment and SelfCog performance

For each subtest, we measured both accuracy (number of correct responses) and the mean response time (RT) of correct answers. The motor subtest, with minimal cognitive load, measures the motor RT to press a button. To ensure this point, we showed that the SelfCog motor RT of Huntington’s disease patients correlated with UHDRS motor scores (TMS: *r* = 0.28; *P* = 0.02 as well as the TMS finger–hand: *r* = 0.39; *P* = 0.001). To calculate cognitive processing times at each assessment, we subtracted the mean motor RT from the mean RT of correct responses of each cognitive subtest. To deal with speed–accuracy trade-off^30^—the balance between making decisions slowly with high accuracy and quickly with high error rate—we calculated the Inverse Efficiency Score (IES)^31^ for each cognitive function. IES consists of the mean cognitive RT (in seconds) divided by the test accuracy, a higher IES indicating a lower performance. We also calculated the global cognitive IES (mean of all cognitive IES).

Missing data in classical cognitive paper-and-pencil tests were imputed using 10 iterations of the non-parametric random forest imputation algorithm^32^ (Supplementary Table 4). The comparison of performance on paper-and-pencil and SelfCog tests between healthy controls and Huntington’s disease participants at M0 was assessed using linear models. The associations between SelfCog and classical scores in Huntington’s disease participants at M0 and the reliability of these measures in all participants between M0 and M1 evaluations were calculated using Spearman’s correlation coefficients and false discovery rate (FDR)–corrected *P*-values. We also used linear mixed-effects models to assess both retest effect (between M0 and M1) and longitudinal evolution (between M1 and M12). This latter analysis aimed to estimate the cognitive decline while controlling for the familiarization effect.^14^ Individual participants were added as random intercepts. Tukey’s *post hoc* comparisons were performed. We computed repeated-measures Cohen’s *d* to measure effect sizes of cognitive changes between M1 and M12. Bootstrapped confidence intervals were estimated with 5000 bootstrap resamples performed per effect size estimated. Statistical models were adjusted for age, sex, education level and study site.

Data management was conducted, and statistical analyses and graphics were generated with R version 4.0.4 (R Core Team,^33^ RStudio (http://www.rstudio.com/).

#### MRI data acquisition, processing and analysis

The detailed description of MRI acquisition and processing is provided in Supplementary Appendix 2. Images were analysed using tools from the FMRIB Software Library (FSL).^34^ Brain MRI scans were obtained for 51 Huntington’s disease participants at M0 and for 24 at M12 in France and Germany (Supplementary Tables 5 and 6). Structural 3D-T1 data were analysed with the standard (M0) and adapted (for longitudinal analysis) processing stream of FSL–voxel-based morphometry (VBM) (https://fsl.fmrib.ox.ac.uk/fsl/fslwiki/FSLVBM).^34–37^ Diffusion-weighted imaging data were processed and analysed with a tract-based spatial statistics (TBSS) pipeline at baseline^38^ and using an optimized registration approach to register baseline and follow-up fractional anisotropy (FA) images for the longitudinal analysis.^39^ The output used in the statistical longitudinal MRI data analysis consisted of the subtraction of the baseline from the follow-up images to obtain a difference image for each subject for grey matter (GM) volumes and white matter (WM) FA.

We applied a voxel-wise general linear model (GLM) with non-parametric permutation tests (10 000 for FSL–VBM and 5000 for TBSS). We identified the GM or WM areas contributing to the regression models of the SelfCog scores and of the classical test performance at baseline in Huntington’s disease participants. A second regression analysis was performed between the change in SelfCog scores or classical test scores and change in GM volumes or FA in Huntington’s disease participants. Family-wise error correction for multiple comparisons was performed, implementing threshold-free cluster enhancement using a significance threshold of *P* < 0.05. Age at baseline assessment, sex, site, DBS (in case of FSL–TBSS analysis) and also the total intracranial volume (in case of FSL–VBM analysis) were entered as covariates of no interests for all imaging analyses.

The association between longitudinal change in both SelfCog and in the classical tests’ performance (delta M12–M1) and the whole brain atrophy obtained with SIENA was evaluated in Huntington’s disease participants using linear regression. Statistical analyses were adjusted for age, sex, MRI site and DBS.

## Results

### Missing responses

The Supplementary Table 7 showed the mean percentage of missing response for the SelfCog at each session of evaluation (M0, M1 and M12). Although there are higher missing responses in Huntington’s disease participants compared to controls, the mean percentage is below 5%.

### Dual-baseline (M0 and M1) analyses

**Figure 2:**
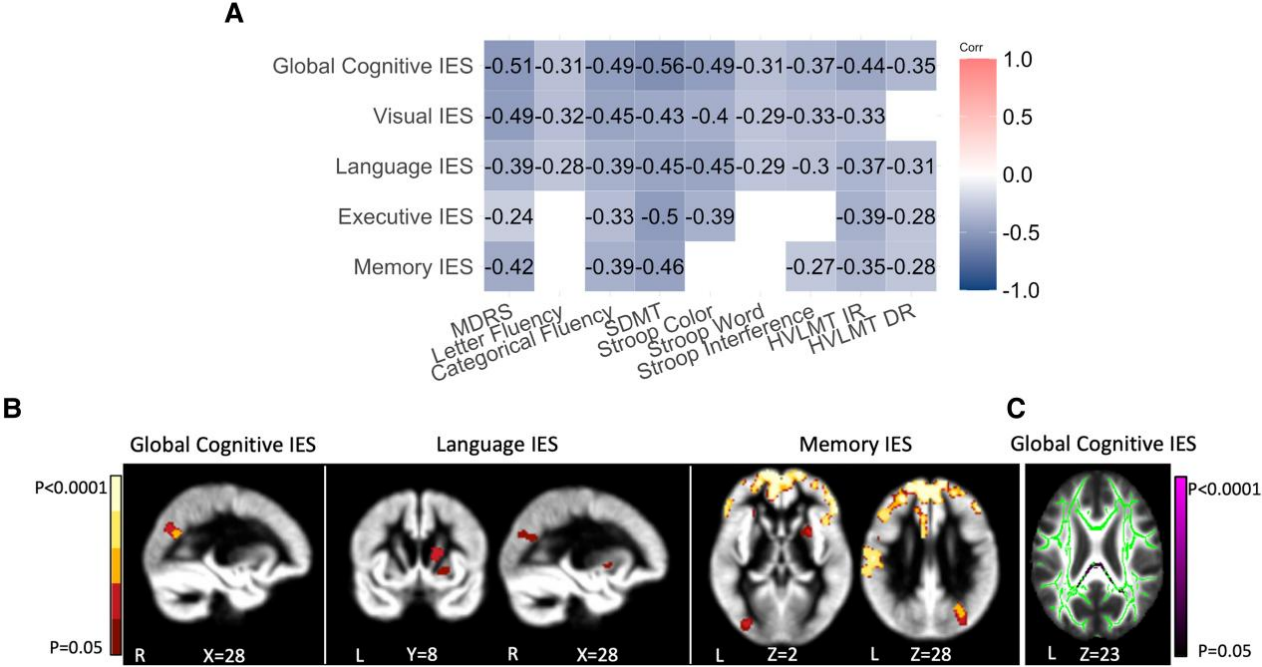
**Correlations between the performance at the SelfCog and the cognitive tests with brain atrophy in Huntington’s disease participants (*n* = 51).** (**A**) Spearman’s correlations between SelfCog’s IES and performance at the classical tests in Huntington’s disease patients. White colour indicates non-significant correlation (*P* < 0.05 after FDR correction). (**B**) VBM results: association between GM volumes and global cognitive, language and memory IES in Huntington’s disease patients. Results are presented at *P* < 0.05 and corrected for multiple comparisons from colour yellow (*P* < 0.0001) to red (*P* < 0.05). Increased global cognitive IES was associated with smaller GM volume in the right occipital cortices. Language IES was associated with GM volume in the right basal ganglia and occipital cortex, in the left temporal cortex and in the bilateral cerebellar lobules VI. Memory IES showed a more widespread negative relationship with GM volume in the bilateral frontal occipital, parietal and temporal areas and in the right basal ganglia. (**C**) TBSS results projected on a template of FA (in green): association between FA and global cognitive IES. Violet colour indicates significant results.

No statistical analysis revealed an effect of the study site in the SelfCog performance (*P* > 0.05 for all tests). Group comparisons for demographics and neurologic evaluations as well as for classical and SelfCog tests at M0 are reported in Tables 1 and 2. All classical and SelfCog tasks distinguished the performance of Huntington’s disease participants from controls. In the SelfCog task, Huntington’s disease participants were slower and less accurate, resulting in higher SelfCog IES compared to controls (all *Ps* < 0.001, Table 1). In Huntington’s disease participants, all SelfCog IES correlated with paper-and-pencil tasks (Fig. 2A). The global cognitive IES correlated with all paper-and-pencil tasks and the UHDRS TFC (*R*^2^ = 0.10, *P* = 0.009), the TMS (*R*^2^ = 0.09, *P* = 0.01) and the functional (*R*^2^ = 0.12, *P* = 0.004) scores.

**Table 2:** Mean cognitive performance at M0 and Pearson’s correlations between M0 and M1

	Controls	Huntington’s disease participants	*P*-values of controls versus Huntington’s disease participants	Pearson’s *r* correlation M0/M1
Global cognitive IES	1.67 ± 0.40 (0.90–2.83)	3.44 ± 1.70 (1.76–12.20)	2.19 e^−11^	0.69
Visual IES	0.86 ± 0.38 (0.28–2.84)	1.89 ± 1.11 (0.28–6.13)	1.03 e^−08^	0.43
Language IES	3.58 ± 1.02 (1.96–6.85)	7.48 ± 4.91 (2.98–38.53)	1.12 e^−07^	0.60
Executive IES	0.776 ± 0.29 (0.36–2.03)	1.56 ± 0.89 (−0.28–4.15)	1.49 e^−08^	0.67
Memory IES	1.46 ± 0.39 (0.80–2.96)	2.84 ± 1.65 (1.01–11.23)	1.05 e^−07^	0.60
MDRS	141.79 ± 2.33 (136.00–146.00)	132.10 ± 7.57 (109.00–144.00)	5.47 e^−15^	0.79
Letter fluency	40.99 ± 10.21 (10.00–67.00)	28.22 ± 8.80 (7.00–47.00)	5.73 e^−12^	0.79
Categorical fluency	21.64 ± 5.84 (11.00–44.00)	14.39 ± 4.67 (6.00–27.00)	1.36 e^−13^	0.76
SDMT	52.08 ± 11.16 (23.00–81.00)	30.32 ± 10.06 (4.00–59.00)	<2 e^−16^	0.94
Stroop colour	78.39 ± 10.53 (56.00–99.00)	49.63 ± 12.40 (27.00–80.00)	<2 e^−16^	0.90
Stroop word	99.72 ± 13.45 (60.00–136.00)	68.12 ± 17.47 (34.00–110.00)	<2 e^−16^	0.88
HVLMT IR	28.50 ± 3.48 (21.00–35.00)	19.55 ± 5.74 (8.00–34.00)	<2 e^−16^	0.81
HVLMT DR	10.31 ± 1.70 (6.00–12.00)	5.87 ± 2.96 (0.00–12.00)	<2 e^−16^	0.82

IES, Inverse Efficiency Score; SDMT, Symbol Digit Modalities Test; MDRS, Mattis Dementia Rating Scale; HVLMT, Hopkins Verbal Learning Memory Test; IR, immediate recall; DR, delayed recall.

Significant relationships between the SelfCog IES and brain imaging data are displayed in Fig. 2B (GM analysis) and in Fig. 2C (WM analysis) and in Table 3. The associations between SelfCog accuracy and RT and paper-and-pencil tests are presented in Supplementary Fig. 1 and Supplementary Table 8 (GM analysis) and Supplementary Fig. 2 and Supplementary Table 9 (WM analysis). Higher global cognitive IES was associated with smaller GM volumes in the right cuneus and right middle/superior occipital cortices as well as with lower FA in the posterior part of the corpus callosum. Language IES was associated with GM volumes in the right basal ganglia (caudate, putamen, pallidum), in the right cuneus, in the right middle/superior occipital cortices and in the left middle/superior temporal cortices and in the left and right cerebellar lobules VI. Language accuracy showed a positive association with GM volumes from extended regions of the basal ganglia, occipital, parietal, temporal and cerebellar cortices. Memory IES showed a negative relationship with GM volumes from extended regions of the bilateral frontal areas, the right basal ganglia (putamen, pallidum) and the bilateral occipital, parietal and temporal cortices. Memory accuracy was associated with GM volumes in the left frontal areas and left inferior/middle occipital cortices.

**Table 3:** Clusters showing decreased grey matter integrity associated with cognitive scores in Huntington’s disease participants at M0

GM clusters	MNI coordinates			Cluster size	Corrected *P*
	*x*	*y*	*z*		
Global—IES					
R middle occipital lobe	28	−68	24	192	0.026
Language—IES					
R caudate	12	8	12	267	0.031
R middle occipital lobe	28	−68	24	71	0.043
L cerebellum (6)	−26	−56	−28	50	0.04
R cerebellum (6)	32	−54	−30	38	0.041
L middle temporal lobe	−52	−52	16	18	0.047
Memory—IES					
L and R medial superior frontal lobe	−6	60	8	21 807	0.002
R middle occipital lobe	36	−72	38	884	0.023
L middle occipital lobe	−34	−84	2	254	0.037
R precuneus	2	−54	60	211	0.042
R putamen	28	8	0	153	0.037
L middle occipital lobe	−28	−92	16	34	0.047
L lingual	−28	−60	−4	31	0.043
L fusiform g	−36	−70	−16	24	0.047

Cluster size, number of voxels, L, left; R, right; g, gyrus. Grey matter clusters significantly associated with cognitive scores (*P*_TFCE_*<*0.05). Coordinates indicate the location of the cluster peak in Montreal Neurological Institute (MNI) convention.

Concerning classical paper-and-pencil tests, the MDRS scores were positively associated with GM volumes in the basal ganglia (caudate, putamen, pallidum) and in the occipital, parietal and temporal cortices. The colour Stroop scores were associated with GM volumes in the occipital, parietal and basal ganglia. The Stroop interference and SDMT scores were associated with GM volumes in the inferior left temporal and occipital cortices, whereas GM volumes in the left basal ganglia were associated with the SDMT only. The immediate recall (IR) of the Hopkins Memory Test was positively associated with GM volumes in the bilateral frontal areas.

The TBSS regressions analysis demonstrated an association of lower FA with higher global IES in the posterior part of the corpus callosum (Table 3). Lower FA in these WM areas was also associated with lower performance on paper-and-pencil tests (MDRS; Stroop—colour and interference, SDMT, Hopkins RI and RD) with additional anterior inter-hemispheric lower FA associated with the MDRS and the colour part of the Stroop (Supplementary Table 9).

Test–retest reliability between M0 and M1 ranged for SelfCog IES from *r* = 0.34 to *r* = 0.60 (Supplementary Table 10). Most tests showed an improvement of performance at M1 compared to M0 in controls and Huntington’s disease participants (Supplementary Fig. 3), with the exception of IES in controls for which the confidence intervals included zero.

### Longitudinal (M1–M12) analysis

In contrast to controls [all *Ps* > 0.05 except for the composite Unified Huntington’s Disease Rating Scale (cUHDRS): <ι>β</i>=0.53, *SE* = 0.22, *P* < 0.02, which improves], Huntington’s disease participants’ functional and motor capacities declined over a year as well as their cUHDRS score (TFC: <ι>β</i>=−0.85, *SE* = 0.16, *P* < 0.0001, FAS: <ι>β</i>=−1.36, *SE* = 0.32, *P* = 0.0001, TMS: *P* = 0.05; <ι>β</i>=2.81, *SE* = 1.04, *P* < 0.01; cUHDRS: <ι>β</i>=−0.85, SE = 0.19, *P* < 0.0001).

**Figure 3:**
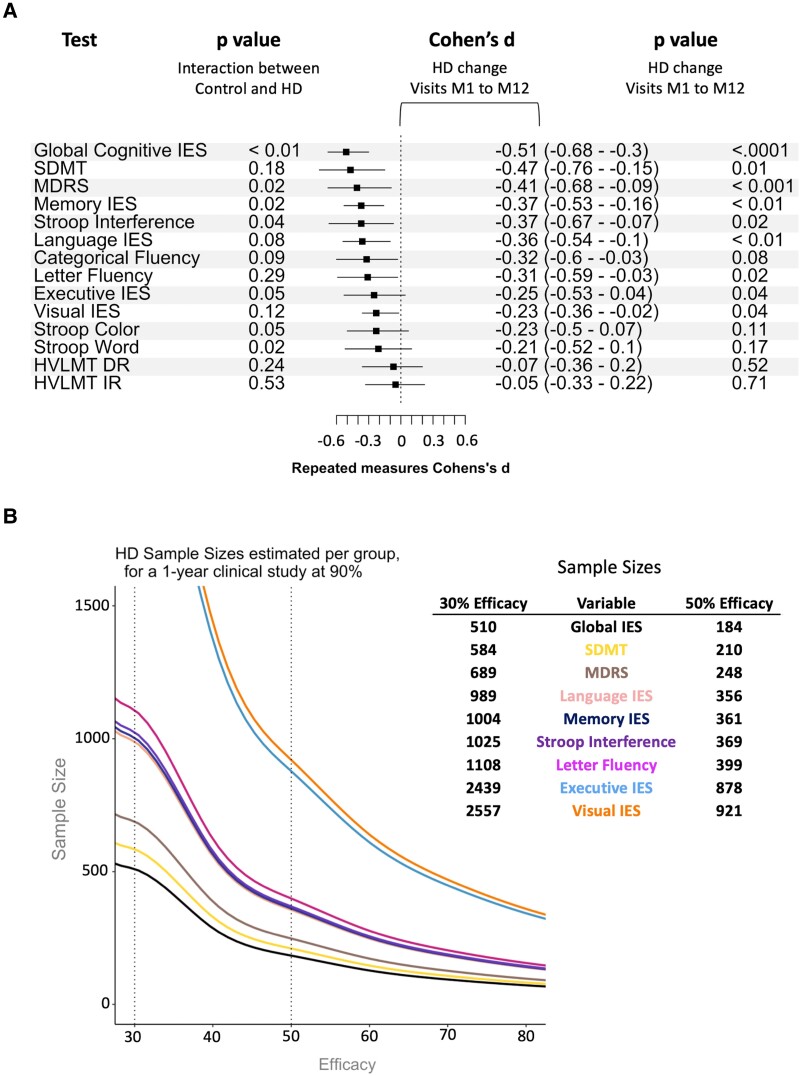
**Effect sizes and Huntington’s disease sample size estimations at a power of 90%.** Fifty-one Huntington’s disease and 40 control participants were included in this analysis. (**A**) Longitudinal Cohen’s *d* effect sizes and 95% confidence intervals for each cognitive test between baseline and Month 1 (M1) and between M1 and M12 evaluations in 51 Huntington’s disease patients. (**B**) Huntington’s disease sample size estimations at a power of 90% at 30 and 50% effectiveness. IES, Inverse Efficiency Score; HVLT, Hopkins Verbal Memory Test; SDMT, Symbol Digit Modality Test.

Cognitive decline over a year was detected using paper-and-pencil tasks such as Stroop interference (*P* = 0.02) and MDRS (*P* < 0.001) (Fig. 3A, Supplementary Tables 11 and 12) in Huntington’s disease participants but not in controls (for which performance in the Stroop word reading task even improved, *P* = 0.05). All IES increased in Huntington’s disease participants (*Ps* < 0.05, Fig. 3A) but remained stable in controls (all *Ps* >0.05). Changes in global cognitive IES, executive IES, memory IES and scores of the MDRS and the three tasks of the Stroop test were higher in Huntington’s disease participants than in controls (*P* < 0.05). *Post hoc* analyses using longitudinal mixed models showed that there was no significant interaction between baseline DBS and time.

In tests showing a decline over a year, the global cognitive IES had the largest effect size (Cohen’s *d*) and was associated with the smallest estimated sample size required for a clinical trial (Fig. 3A and B). Effect sizes estimated for memory and executive IES were comparable to those of MDRS and Stroop interference.

Brain atrophy progression was associated with language (*P* = 0.03) and global (*P* = 0.003) IES (Supplementary Fig. 4) and language (*P* = 0.006) and visual (*P* = 0.015) accuracy. In our cohort, the rate of brain atrophy was ∼1% per year. Only language IES was associated with change in FA in the posterior part of the corpus callosum in the right hemisphere (Supplementary Fig. 4). We did not observe any significant association between changes in scores on the SelfCog or classical paper-and-pencil tasks and changes in GM volumes or FA.

## Discussion

Using the methodological approach derived from neuroscience concepts, we have developed and assessed prospectively a brief digitized cognitive battery named SelfCog in four sites in three different languages (French, English and German) in Huntington’s disease. The SelfCog detected cognitive decline over 1 year with greater sensitivity than classical paper-and-pencil cognitive tests with good psychometric properties of SelfCog including construct validity and reliability. Lower performance was associated with GM atrophy and FA decrease coherent with cerebral damage of the condition. These results make the SelfCog an excellent candidate for use in future therapeutic trials and in clinical practice. In addition, it advocates for the clinical cognitive field move towards a larger application of the neuroscience methods.

The SelfCog offers a conceptual break into the neuropsychological landscape by its original design inspired by the experimental cognitive neuroscience principles. Functional MRI experiments, as well as experimental cognition, classically equalize all the inputs (stimuli) and outputs (responses) when comparing conditions except the one of interest. This allows functions to be compared with each other, with a single paradigm.^21^ In contrast, most cognitive paper-and-pencil tasks use a variety of sensory-motor modalities of the stimuli or modes of responses, such as word reading in the Stroop test and matching written figures to numbers in the SDMT for example.^8^ This makes it impossible to disentangle which function is impaired between reading, visual perception, speech and motor response in such example. The variety of impairments in cognitive functions in Huntington’s disease has shown that exhaustive evaluation should take into account at least language, executive functions, memory, visuo-perceptive function like in the CAPIT-HD, the CANTAB or the Huntington’s Disease—Cognitive Assessment Battery (HD-CAB).^12^ However, none of them was able to provide a unique composite end-point reflecting the global cognitive capacity. Additionally, neuroscience can distinguish the stages of perception, function processing, decision-making and motor responses to analyse the function of interest in statistical accumulation models.^40^ These models are not applicable in the type of neuropsychological assessment we provide here, given the low amount of data by individuals. An alternative consists in neutralizing the steps common to each condition by subtracting the motor condition which also includes a perceptual and decision phase and by analysing the remaining components, namely, language, memory, visuo-spatial processing and executive functions. This is crucial as motor and cognitive capacities may evolve differently following intracerebral interventions^41^ or pharmaceutical treatment.^42^ Motor decline might mask possible benefits in clinical trials towards improving attention,^43^ executive functions^44^ or global cognitive functioning.^45,46^ The response times obtained from the motor subtest being significantly correlated with the Total Motor Score^8^ in the participants make the motor score a valid motor baseline.

An additional principle of the neuroscience experiments is the randomization.^47^ The sequence of the stimuli combined with the participants’ fatigue may induce a bias. In addition, participants in clinical studies are exposed subsequent times to evaluation. Therefore, the potential retest effect is a major concern and might be decomposed between a familiarization effect and a learning effect.^13^ The familiarization effect is inherent to any human activity; the second time of acting is always easier than the first one. The learning effect consists of the recall of the stimuli of the experiments. In clinical trials, some participants may have been exposed previously to the paper-and-pencil tasks, while for others, the tasks are totally novel. Here, to reduce the inherent discrepancy between previously exposed (familiarized) and never exposed (unfamiliarized) patients, we proposed a sequence of evaluation with M0 used to maximize the familiarization at M1. We thus used a M0–M1 dual baseline to control for the familiarization effect and measured longitudinal decline by comparing the M1–M12 assessments.^12,14^ To avoid the learning effect, we conceived a stimuli randomization algorithm selecting a set of 200 pictures appropriate for each task among the 585 of our pictures’ bank; a new form is generated for each assessment while keeping the task demand constant. To our knowledge, this strategy has never been applied to cognitive clinical assessments. As a result, we were able to show that the SelfCog is less prone to test–retest (or learning) effects than the paper-and-pencil test. This was especially true for control participants who showed none of the familiarization effects that are often observed between the first and second visits. Nor did they show a learning effect, as evidenced by the lack of improvement in any subtasks over 12 months, even in the memory subtask. All these design characteristics provide a remarkable discriminant and convergent validities of individual cognitive SelfCog’s scores in Huntington’s disease and healthy control participants with coherent correlations of performance of each cognitive performance in the SelfCog and classical paper-and-pencil tests.

The translational perspective is a crucial issue in the development of tests adapted to clinical trials, as demonstrated by the success of the strategy under the development of the CANTAB.^48^ Animal experiments simplify instructions and response mode as much as possible with non-verbal inputs and outputs.^49^ The use of non-verbal stimuli in the SelfCog could provide an opportunity for back-translation to pre-clinical studies (except for the language subtest), which in turn has the potential to enhance the validity of animal models. This is crucial for therapeutics development programmes, which could benefit from the use of tasks with analogues in rodents, non-human primates and humans.^49,50^

We have chosen to develop a digitized test to meet the requirements of cognitive end-points needed to monitor the evolution of symptoms in clinical trials. The review of Mestre and collaborators^5^ pointed out that none of the cognitive scales used in Huntington’s disease met the criteria for a ‘recommended’ status. In contrast with classical paper-and-pencil cognitive tests, the digitized SelfCog battery provides a precise recording of accuracy and RT, which enhances the cognitive evaluation. The speed–accuracy trade-off makes it difficult to interpret between the RT slow-down to avoid errors and RT increase without any concern on their accuracy. To address the speed–accuracy trade-off problem, we combined response times and accuracy in a single score (IES), which showed greater sensitivity to change than the two measures separately.^16,51^ We then assessed the sensitivity of the SelfCog to measure cognitive decline by conducting a longitudinal study in a subset of participants, as cross-sectional studies often lead to overestimation of the sensitivity to detect a longitudinal change.^23^ IES of the SelfCog estimated cognitive decline with the largest longitudinal effect sizes compared to classical cognitive tests. This decline was independent from study site or mother tongue. IES also showed reduced longitudinal intra- and inter-subject variability as evidenced by the relatively small confidence intervals of their effect sizes. The global cognitive IES meets the requirement to provide a single primary end-point to be used in clinical trials.^4,12^ Rather than providing an *ad hoc* construct like the sum of the cognitive assessments of the UHDRS which overweight the Stroop test,^8^ it combines with equal weight language, executive, visual and memory functions. This allows intra- and inter-comparison of participants’ cognitive performance over time, regardless of their cognitive profile. Such balance presumably explains its largest effect size compared to all other cognitive evaluation within the study. The global SelfCog IES correlated with the TFC functional score, which denotes its ecological validity.^6^ It may predict the participant’s capacity to behave in daily life and highlights its relevance in Huntington’s disease. The MDRS, SDMT and Stroop tests also well captured cognitive change. However, the MDRS is a multidimensional test of limited use nowadays for monitoring Huntington’s disease symptoms because it suffers from a floor effect, of translational difficulties and of too long assessment (taking around 30 min to complete).^5^ Despite its efficacy in monitoring the disease progression, the Stroop test is hampered by reading capacities and cross-linguistic differences which may explain the lack of systematic decline in longitudinal studies.^12^ Finally, the SDMT, considered as one of the best markers of decline in Huntington’s disease, combines visual, executive and motor functions, thus precluding their disentangling.^52^

Finally, a key aspect to cognitive assessment is their consistency with the brain deterioration as shown by brain imaging. Both the SelfCog and the paper-and-pencil test scores were associated with the brain atrophy and the structural impairment of the white matter pathways. This adds evidence for the convergent validity of the battery. For example, the scores of the memory subtest of the SelfCog and the recall of the HVLMT were both associated with grey matter volumes of the frontal regions involved in memory performance. The striatum volume or the FA in the splenium of the corpus callosum^53,54^ was associated with global IES, the memory IES and the language IES scores and accuracy of the SelfCog. In the longitudinal analysis, the rate of atrophy was associated with the evolution of the global cognitive and visual accuracy and both language IES and accuracy. The rate of brain atrophy of around 1% per year in Huntington’s disease is similar to that reported by others.^23^ Likewise, the change in fractional anisotropy in the splenium of the corpus callosum over time was associated with the language decline in the SelfCog, the corpus callosum being known as particularly sensitive to early damage in Huntington’s disease.^39,55–57^

Our results support previous associations between reduced grey matter volumes in the occipital lobe and cognitive impairment in Huntington’s disease.^55,58^ However, at baseline and longitudinally, the global cognitive score was not associated with striatal volumes. This might reflect that the cognitive score reflects all cognitive functions including the ones connected to the striatum but also to the cortex (impaired even at the premanifest stages). As mentioned earlier, whereas the striatum is the best measurable marker of the disease evolution as it requires less participants than the other markers to demonstrate a decline, it does not exclude that in the other structure the evolution is not linear and may be asynergic. The full impact of the disease on the brain including the cortex might be more difficult to capture. In addition, the number of available MRI scanning was lower to the behavioural scores, and such discrepancy might have blurred the picture.

## Conclusion

Overall, SelfCog provides a good balance of brevity, standardization and comprehensiveness for monitoring cognitive functions in Huntington’s disease. It confirms the usefulness of neuroscience principles in moving beyond current digitized assessments based on paper-and-pencil task translation. The SelfCog composite global cognitive score could be used as an end-point in clinical trials aiming to slow down cognitive impairment progression or as a variable of diagnostic or prognostic interest in the disease combined with motor and psychiatric variables and possibly combined with neuroimaging or biofluid biomarkers. In addition, SelfCog allows a specific monitoring of each cognitive function and thus allows the detection of asynchronous evolutions related to the variability of the disease or to the heterogeneity of the impact of Huntington’s disease treatments.^22^ There are some limitations in this study. One of them is the relatively small cohort size although being able to detect the cognitive decline. Future studies need to confirm our results in new cohorts of HD participants. Despite this, the finding of large effect sizes and associations with brain structure shows the potential of the task in assessing and monitoring cognitive function. The coding of the stimuli using a randomization algorithm allows for good performance consistency between French, English and German and makes translation into other languages plausible within a reasonable time frame. Moreover, in most clinical trials targeting participants before the onset of the disease, it would be important to verify its validity in premanifest participants. Future studies should also examine its validity in late-stage HD participants and its suitability for a home-based follow-up of individuals with HD. Currently, the SelfCog is likely to allow both screening whether patients need a neuropsychological consultation and repeated assessment to monitor subtle cognitive change between hospital visits and in clinical trials. By addressing the concern of the comparison of the progression of the different cognitive impairments while controlling the motor component, the interpretation of the performance will be simplified. Recognizing that Huntington’s disease exhibits the triad of symptoms observed in most NDDs,^24^ with progressive deterioration of cognitive, psychiatric and motor functions, excellent applicability to other diseases can be assumed. Future studies should assess the capacity of the SelfCog to be sensitive to the different neurodegenerative processes and to disease stage.

## Supplementary Material

fcad043_Supplementary_Data

## Data Availability

The anonymized datasets of the current study are available from the corresponding author on reasonable request.

## Abbreviations

CANTAB =: Cambridge Neuropsychological Test Automated Battery
cUHDRS =: composite Unified Huntington’s Disease Rating Scale
DAT =: digitized arithmetic task
DBS =: disease burden score
FA =: fractional anisotropy
FDR =: false discovery rate
FSL =: FMRIB Software Library
GLM =: general linear model
GM =: grey matter
HD-CAB =: Huntington’s Disease—Cognitive Assessment Battery
HVLMT =: Hopkins Verbal Learning Memory Test
IES =: Inverse Efficiency Score
MDRS =: Mattis Dementia Rating Scale
RT =: response time
SDMT =: Symbol Digit Modalities Test
TBSS =: tract-based spatial statistics
TMT =: Trail Making Test
UHDRS =: Unified Huntington’s Disease Rating Scale
VBM =: voxel-based morphometry
WM =: white matter

## References

[1] Pasquier F . Early diagnosis of dementia: Neuropsychology. J Neurol. 1999;246:6–15. 9987708 10.1007/s004150050299

[2] Tabrizi SJ , Ghosh R, Leavitt BR. Huntingtin lowering strategies for disease modification in Huntington’s disease. Neuron. 2019;101:801–819. 30844400 10.1016/j.neuron.2019.01.039

[3] Dorsey ER , Papapetropoulos S, Xiong M, Kieburtz K. The first frontier: Digital biomarkers for neurodegenerative disorders. Digit Biomark. 2017;1:6–13. 32095743 10.1159/000477383 PMC7015357

[4] Schobel SA , Palermo G, Auinger P, et al Motor, cognitive, and functional declines contribute to a single progressive factor in early HD. Neurology. 2017;89:2495–2502. 29142089 10.1212/WNL.0000000000004743 PMC5729794

[5] Mestre TA , Bachoud-Lévi AC, Marinus J, et al Rating scales for cognition in Huntington’s disease: Critique and recommendations. Mov Disord. 2018;33:187–195. 29278291 10.1002/mds.27227 PMC10080408

[6] Howieson D . Current limitations of neuropsychological tests and assessment procedures. Clin Neuropsychol. 2019;33:200–208. 30608020 10.1080/13854046.2018.1552762

[7] Corey-Bloom J , Williams ME, Beltran-Najera I, et al Central cognitive processing speed is an early marker of Huntington’s disease onset. Mov Disord Clin Pract. 2021;8:100–105. 33426164 10.1002/mdc3.13121 PMC7781078

[8] Huntington Study Group . Unified Huntington’s disease rating scale: Reliability and consistency. Mov Disord. 1996;11:136–142. 8684382 10.1002/mds.870110204

[9] Harris KL , Kuan WL, Mason SL, Barker RA. Antidopaminergic treatment is associated with reduced chorea and irritability but impaired cognition in Huntington’s disease (Enroll-HD). J Neurol Neurosurg Psychiatry. 2020;91:622–630. 32229581 10.1136/jnnp-2019-322038 PMC7279191

[10] Brandt J . The Hopkins Verbal Learning Test: Development of a new memory test with six equivalent forms. Clin Neuropsychol. 1991;5:125–142.

[11] Rieu D , Bachoud-Lévi AC, Laurent A, Jurion E, Dalla Barba G. Adaptation française du « Hopkins Verbal Learning Test ». Rev Neurol (Paris). 2006;162:721–728. 16840980 10.1016/s0035-3787(06)75069-x

[12] Stout JC , Queller S, Baker KN, et al HD-CAB: A cognitive assessment battery for clinical trials in Huntington’s disease^1,2,3^. Mov Disord. 2014;29:1281–1288. 25209258 10.1002/mds.25964

[13] Bachoud-Lévi AC , Maison P, Bartolomeo P, et al Retest effects and cognitive decline in longitudinal follow-up of patients with early HD. Neurology. 2001;56:1052–1058. 11320178 10.1212/wnl.56.8.1052

[14] Schramm C , Katsahian S, Youssov K, et al How to capitalize on the retest effect in future trials on Huntington’s disease. PloS One. 2015;10:e0145842. 26714284 10.1371/journal.pone.0145842 PMC4703129

[15] Schneider LS , Goldberg TE. Composite cognitive and functional measures for early stage Alzheimer’s disease trials. Alzheimers Dement Diagn Assess Dis Monit. 2020;12:e12017. 10.1002/dad2.12017 PMC7233425 32432155

[16] Lunven M , Hamet Bagnou J, Youssov K, et al Cognitive decline in Huntington’s disease in the Digitalized Arithmetic Task (DAT). PloS One. 2021;16:e0253064. 34424902 10.1371/journal.pone.0253064 PMC8382187

[17] Langley C , Gregory S, Osborne-Crowley K, et al Fronto-striatal circuits for cognitive flexibility in far from onset Huntington’s disease: Evidence from the Young Adult Study. J Neurol Neurosurg Psychiatry. 2021;92:143–149. 33130575 10.1136/jnnp-2020-324104 PMC7841479

[18] Boada M , Rodrigo A, Jessen F, et al Complementary pre-screening strategies to uncover hidden prodromal and mild Alzheimer’s disease: Results from the MOPEAD project. Alzheimers Dement. 2022;18:1119–1127. 34310061 10.1002/alz.12441 PMC9290633

[19] Ra B , Sl M, Tp H, et al The long-term safety and efficacy of bilateral transplantation of human fetal striatal tissue in patients with mild to moderate Huntington’s disease. J Neurol Neurosurg Psychiatry. 2013;84:657–6665. 23345280 10.1136/jnnp-2012-302441 PMC3646287

[20] Lenehan ME , Summers MJ, Saunders NL, Summers JJ, Vickers JC. Does the Cambridge Automated Neuropsychological Test Battery (CANTAB) distinguish between cognitive domains in healthy older adults? Assessment. 2016;23:163–172. 25882162 10.1177/1073191115581474

[21] Amaro E , Barker GJ. Study design in fMRI: Basic principles. Brain Cogn. 2006;60:220–232. 16427175 10.1016/j.bandc.2005.11.009

[22] Tabrizi SJ , Scahill RI, Owen G, et al Predictors of phenotypic progression and disease onset in premanifest and early-stage Huntington’s disease in the TRACK-HD study: Analysis of 36-month observational data. Lancet Neurol. 2013;12:637–649. 23664844 10.1016/S1474-4422(13)70088-7

[23] Tabrizi SJ , Reilmann R, Roos RAC, et al Potential endpoints for clinical trials in premanifest and early Huntington’s disease in the TRACK-HD study: Analysis of 24 month observational data. Lancet Neurol. 2012;11:42–53. 22137354 10.1016/S1474-4422(11)70263-0

[24] Rosser AE , Busse ME, Gray WP, et al Translating cell therapies for neurodegenerative diseases: Huntington’s disease as a model disorder. Brain. 2022;145:1584. 35262656 10.1093/brain/awac086 PMC9166564

[25] Shoulson I . Huntington disease: Functional capacities in patients treated with neuroleptic and antidepressant drugs. Neurology. 1981;31:1333–1333. 6125919 10.1212/wnl.31.10.1333

[26] Mattis S . Mental status examination for organic mental syndrome in the elderly patients. In: Bellack L and Karusu TB, editors. Geriatric psychiatry. Grune & Stratton, 1976. p. 77–121.

[27] Penney JB , Vonsattel JP, MacDonald ME, Gusella JF, Myers RH. CAG repeat number governs the development rate of pathology in Huntington’s disease. Ann Neurol. 1997;41:689–692. 9153534 10.1002/ana.410410521

[28] Reilmann R , Schubert R. Motor outcome measures in Huntington disease clinical trials. Handb Clin Neurol. 2017;144:209–225. 28947119 10.1016/B978-0-12-801893-4.00018-3

[29] Butters N , Wolfe J, Granholm E, Martone M. An assessment of verbal recall, recognition and fluency abilities in patients with Huntington’s disease. Cortex. 1986;22:11–32. 2940074 10.1016/s0010-9452(86)80030-2

[30] Liesefeld HR , Janczyk M. Combining speed and accuracy to control for speed-accuracy trade-offs(?). Behav Res Methods. 2019;51:40–60. 30022459 10.3758/s13428-018-1076-x

[31] Bruyer R , Brysbaert M. Combining speed and accuracy in cognitive psychology: Is the inverse efficiency score (IES) a better dependent variable than the mean reaction time (RT) and the percentage of errors (PE)? Psychol Belg. 2013;51:5–13.

[32] Stekhoven DJ , Bühlmann P. MissForest--non-parametric missing value imputation for mixed-type data. Bioinformatics. 2012;28:112–118. 22039212 10.1093/bioinformatics/btr597

[33] R Core Team . 2020. European Environment Agency. Accessed 3 September 2021. https://www.eea.europa.eu/data-and-maps/indicators/oxygen-consuming-substances-in-rivers/r-development-core-team-2006.

[34] Smith SM , Jenkinson M, Woolrich MW, et al Advances in functional and structural MR image analysis and implementation as FSL. NeuroImage. 2004;23(Supplement 1):S208–S219. 15501092 10.1016/j.neuroimage.2004.07.051

[35] Douaud G , Smith S, Jenkinson M, et al Anatomically related grey and white matter abnormalities in adolescent-onset schizophrenia. Brain. 2007;130(Pt 9):2375–2386. 17698497 10.1093/brain/awm184

[36] Good CD , Johnsrude IS, Ashburner J, Henson RN, Friston KJ, Frackowiak RS. A voxel-based morphometric study of ageing in 465 normal adult human brains. NeuroImage. 2001;14(1 Pt 1):21–36. 11525331 10.1006/nimg.2001.0786

[37] Thomas AG , Marrett S, Saad ZS, Ruff DA, Martin A, Bandettini PA. Functional but not structural changes associated with learning: An exploration of longitudinal voxel-based morphometry (VBM). NeuroImage. 2009;48:117–125. 19520171 10.1016/j.neuroimage.2009.05.097 PMC2981435

[38] Smith SM , Jenkinson M, Johansen-Berg H, et al Tract-based spatial statistics: Voxelwise analysis of multi-subject diffusion data. NeuroImage. 2006;31:1487–1505. 16624579 10.1016/j.neuroimage.2006.02.024

[39] Pflanz CP , Charquero-Ballester M, Majid DSA, et al One-year changes in brain microstructure differentiate preclinical Huntington’s disease stages. NeuroImage Clin. 2020;25:102099. 31865023 10.1016/j.nicl.2019.102099 PMC6931230

[40] Wiecki TV , Antoniades CA, Stevenson A, et al A computational cognitive biomarker for early-stage Huntington’s disease. PloS One. 2016;11:e0148409. 26872129 10.1371/journal.pone.0148409 PMC4752511

[41] Bachoud-Lévi AC , Gaura V, Brugières P, et al Effect of fetal neural transplants in patients with Huntington’s disease 6 years after surgery: A long-term follow-up study. Lancet Neurol. 2006;5:303–309. 16545746 10.1016/S1474-4422(06)70381-7

[42] Murman DL , Giordani B, Mellow AM, et al Cognitive, behavioral, and motor effects of the NMDA antagonist ketamine in Huntington’s disease. Neurology. 1997;49:153–161. 9222184 10.1212/wnl.49.1.153

[43] Beglinger LJ , Adams WH, Paulson H, et al Randomized controlled trial of atomoxetine for cognitive dysfunction in early Huntington disease. J Clin Psychopharmacol. 2009;29:484–487. 19745649 10.1097/JCP.0b013e3181b2ac0a PMC3806326

[44] Beglinger LJ , Adams WH, Langbehn D, et al Results of the citalopram to enhance cognition in Huntington disease trial. Mov Disord. 2014;29:401–405. 24375941 10.1002/mds.25750 PMC3960314

[45] Huntington Study Group HART Investigators . A randomized, double-blind, placebo-controlled trial of pridopidine in Huntington’s disease. Mov Disord. 2013;28:1407–1415. 23450660 10.1002/mds.25362

[46] HORIZON Investigators of the Huntington Study Group and European Huntington’s Disease Network* . A randomized, double-blind, placebo-controlled study of latrepirdine in patients with mild to moderate Huntington disease. JAMA Neurol. 2013;70:25–33. 23108692 10.1001/2013.jamaneurol.382

[47] Kessels RPC . Improving precision in neuropsychological assessment: Bridging the gap between classic paper-and-pencil tests and paradigms from cognitive neuroscience. Clin Neuropsychol. 2019;33:357–368. 30394172 10.1080/13854046.2018.1518489

[48] Nithianantharajah J , McKechanie AG, Stewart TJ, et al Bridging the translational divide: Identical cognitive touchscreen testing in mice and humans carrying mutations in a disease-relevant homologous gene. Sci Rep. 2015;5:14613. 26423861 10.1038/srep14613 PMC4589696

[49] Talpos J , Steckler T. Touching on translation. Cell Tissue Res. 2013;354:297–308. 23949375 10.1007/s00441-013-1694-7

[50] Bussey T , Holmes A, Lyon L, et al New translational assays for preclinical modelling of cognition in schizophrenia: The touchscreen testing method for mice and rats. Neuropharmacology. 2012;62:1191–1203. 21530550 10.1016/j.neuropharm.2011.04.011 PMC3168710

[51] Statsenko Y , Habuza T, Gorkom KNV, Zaki N, Almansoori TM. Applying the inverse efficiency score to visual–motor task for studying speed-accuracy performance while aging. Front Aging Neurosci. 2020;12:574401. 33362528 10.3389/fnagi.2020.574401 PMC7757351

[52] Chen MH , Chiaravalloti ND, Genova HM, Costa SL. Visual and motor confounds on the symbol digit modalities test. Mult Scler Relat Disord. 2020;45:102436. 32750607 10.1016/j.msard.2020.102436

[53] Johnson EB , Ziegler G, Penny W, et al Dynamics of cortical degeneration over a decade in Huntington’s disease. Biol Psychiatry. 2021;89:807–816. 33500176 10.1016/j.biopsych.2020.11.009 PMC7986936

[54] Estevez-Fraga C , Scahill R, Rees G, Tabrizi SJ, Gregory S. Diffusion imaging in Huntington’s disease: Comprehensive review. J Neurol Neurosurg Psychiatry. 2021;92:62–69. 10.1136/jnnp-2020-324377 PMC7803908 33033167

[55] Estevez-Fraga C , Scahill RI, Durr A, et al Composite UHDRS correlates with progression of imaging biomarkers in Huntington’s disease. Mov Disord. 2021;36:1259–1264. 33471951 10.1002/mds.28489

[56] Poudel GR , Stout JC, Domínguez DJF, et al Functional changes during working memory in Huntington’s disease: 30-month longitudinal data from the IMAGE-HD study. Brain Struct Funct. 2015;220:501–512. 24240602 10.1007/s00429-013-0670-z

[57] Rosas HD , Lee SY, Bender AC, et al Altered white matter microstructure in the corpus callosum in Huntington’s disease: Implications for cortical “disconnection”. NeuroImage. 2010;49:2995–3004. 19850138 10.1016/j.neuroimage.2009.10.015 PMC3725957

[58] Martinez-Horta S , Sampedro F, Horta-Barba A, et al Structural brain correlates of dementia in Huntington’s disease. NeuroImage Clin. 2020;28:102415. 32979842 10.1016/j.nicl.2020.102415 PMC7519361

