# Supplementary material for: A new approach to digitized cognitive monitoring: validity of the SelfCog in Huntington’s disease
Source: Brain Commun. 2023 Mar 6;5(2):fcad043. doi: 10.1093/braincomms/fcad043 (PMC10018460; doi:10.1093/braincomms/fcad043)
Supplement: fcad043_Supplementary_Data [file fcad043_supplementary_data.zip › Supplementary_material.pdf]

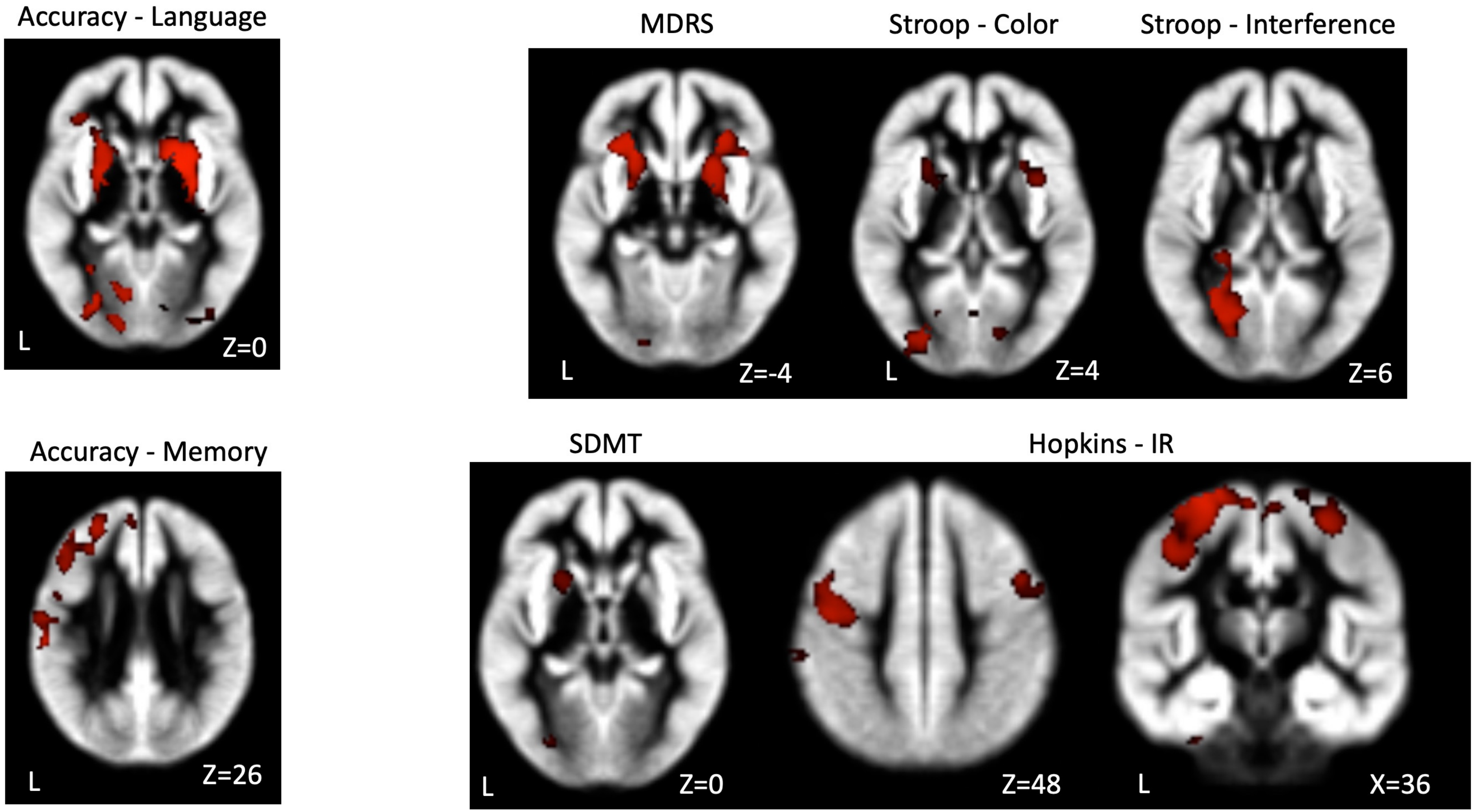

Supplementary Figure 1: VBM results in Huntington's Disease participants (N=51) at baseline

Voxel-wise general linear model with nonparametric permutation tests (10,000).

Association between grey matter volumes and the SelfCog measures (Language and Memory accuracy) and in conventional paper-and-pencil measures (MDRS, Stroop Color, Stroop Interference, SDMT, and Hopkins Verbal Memory Test – Immediate recall). Results (in red) are represented at $p<0.05$ and corrected for multiple comparisons.

MDRS Mattis Dementia Rating Scale, SDMT Symbol Digit Modalities Test, IR Immediate Recall, L left hemisphere

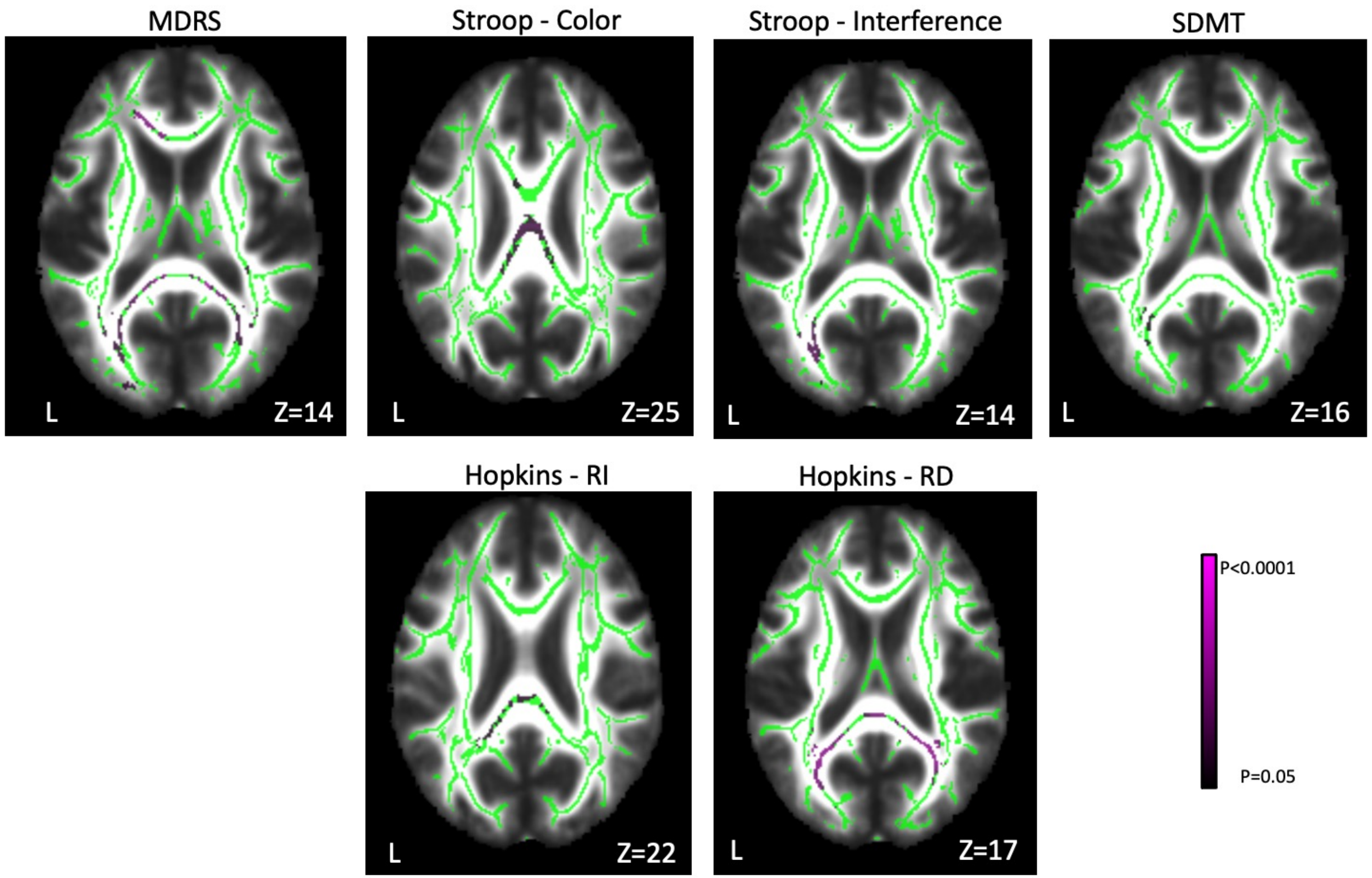

Supplementary Figure 2: TBSS results (in violet) at baseline

Voxel-wise general linear model with nonparametric permutation tests (5,000).

Association in Huntington's Disease participants (N=32) between Fractional Anisotropy and the conventional paper-and-pencil measures (MDRS, Stroop Color, Stroop Interference, SDMT, and Hopkins Verbal Memory Test – Immediate and delayed recalls). Results are represented at $p<0.05$ and corrected for multiple comparisons. All significant results (in violet) are presented on a template of FA (in green).

MDRS Mattis Dementia Rating Scale, SDMT Symbol Digit Modalities Test, IR Immediate Recall, DR Delayed Recall, L left hemisphere

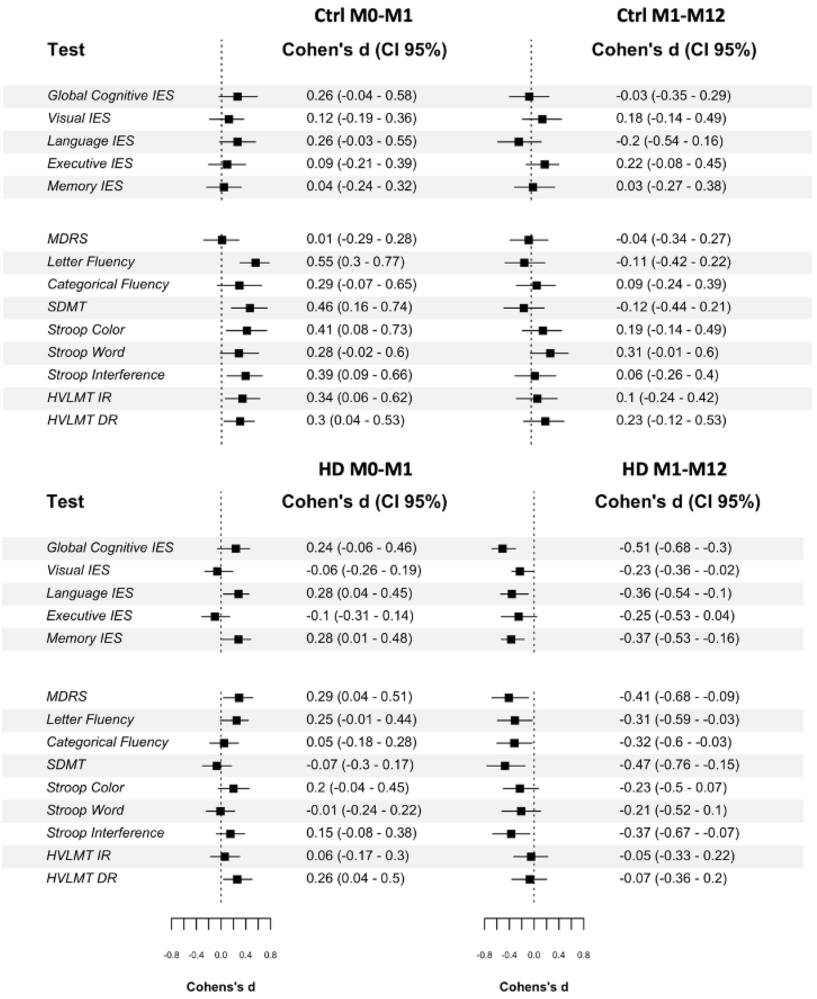

Supplementary Figure 3: Longitudinal Cohen's d effect sizes and 95% confidence intervals for each cognitive test between baseline and month 1 and between M0 and M1 and between M1 and month 12 evaluations in Controls (N=50 and N=40) and HD participants (N= 74 and N=51) respectively.

Bootstrapped confidence intervals were estimated with 5,000 bootstrap resamples performed per effect size estimated. Statistical models were adjusted for age, sex, education level, and study site.

The MDRS effect size for controls is shown in the figure, but it is necessary to clarify that the criteria for inclusion and retention in the study required a score of 136 or higher.

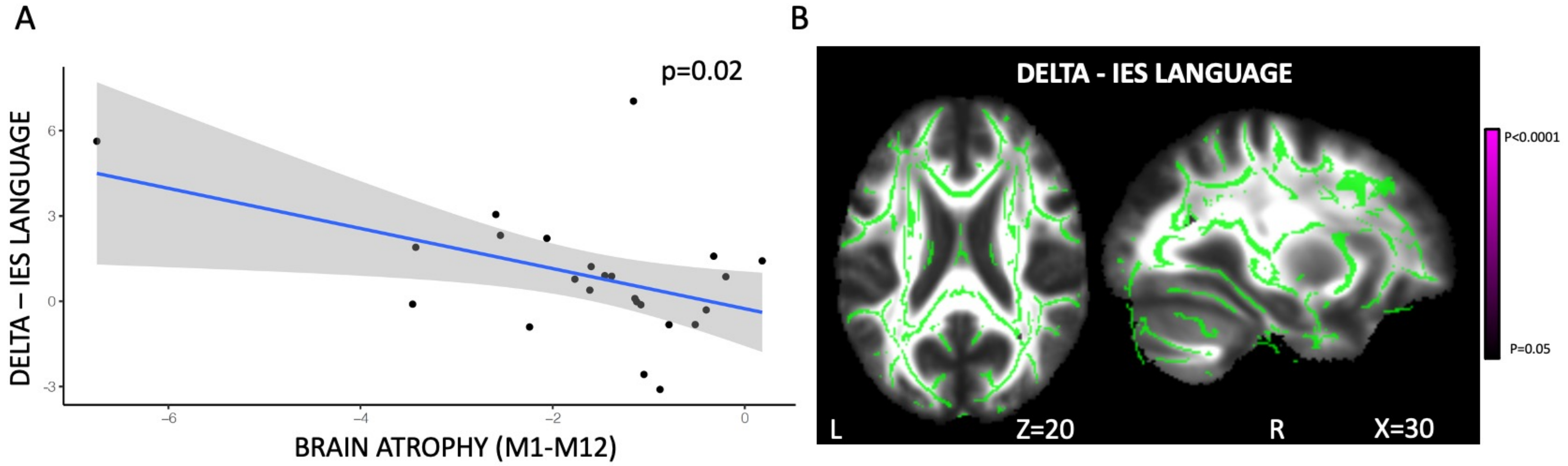

Supplementary Figure 4: Longitudinal neuro-imaging results. A. Association between the delta IES language and brain atrophy over one-year in Huntington's Disease participants (N=24). B. Association of change in IES Language and in Fractional atrophy over one year in Huntington's Disease participants (N=20). All significant results (in violet) are presented on a template of FA (in green).

All the results (in violet) are represented at p<0.05 and corrected for multiple comparisons. We applied a voxel-wise general linear model (GLM) with nonparametric permutation tests for TBSS analysis (5,000).

L left hemisphere, R right hemisphere

Supplementary material

Supplementary Table 1: Demographics for healthy controls and HD patients included in retest-effect analysis

	Control (N=50)	HD (N=74)	p value
**Age**	50.67 (10.35)	52.47 (11.22)	0.37
[Range]	[26.23 - 70.05]	[23.24 - 78.16]	
**Sex**	26F/24M	27F/47M	0.09
**Education**	14.04 (2.84)	14.10 (2.96)	0.92
[Range]	[8.00 - 24.00]	[9.00 - 20.00]	
**Laterality**	1A/5L/44R	0A/7L/67R	0.47
**CAG Repeat**	-	43.66 (3.88)#	
[Range]	-	[38.00 - 62.00]	
**Age At Onset**	-	47.84 (11.06)##	
[Range]	-	[20.02 - 73.83]	
**Disease Duration**	-	4.60 (3.86)##	
[Range]	-	[0.12 - 19.29]	
**DBS**	-	395.39 (102.01)#	
[Range]	-	[130.16 - 674.89]	
**Time between visits 0 and 1**	30.08 (8.14)	34.08 (19.18)	0.17
[Range]	16.00 - 49.00	14.00 - 125.00	

F Female. M Male. R Right. L Left. A Ambidextrous.# 1 missing data point. ## 5 missing data points
Three participants completed the second assessment after a delay (103. 115. and 125 days after the first assessment) due to restrictions brought on by the COVID-19 pandemic.

Supplementary Table 2: Demographics for healthy controls and HD patients included in the longitudinal analysis

	Control (N=40)	HD (N=51)	p value
**Age**	51.94 (10.61)	53.29 (10.62)	0.55
[Range]	[26.98 - 70.97]	[24.23 - 69.99]	
**Sex**	18F/22M	23F/28M	0.99
**Education**	14.08 (2.93)	13.94 (2.93)	0.83
[Range]	[11.00 - 24.00]	[9.00 - 20.00]	
**Laterality**	1A/5L/34R	0A/5L/46R	0.47
**CAG_Repeat**	-	43.75 (4.06)	
[Range]	-	[38.00 - 62.00]	
**AgeAtOnset**	-	47.78 (10.69)	
[Range]	-	[20.02 - 64.54]	
**DiseaseDuration**	-	5.86 (3.98)	
[Range]	-	[0.99 - 20.19]	
**DBS**	-	409.34 (109.99)	
[Range]	-	[137.27 - 699.49]	

F Female. M Male. R Right. L Left. A Ambidextrous.

Supplementary Table 3: Comparison of demographics for heathy and HD patients at baseline according to their participation in the longitudinal follow-up. Statistical analysis did not reveal significant changes between the subjects lost to follow-up and the remaining subjects.

	**All Participants**		**Participants Lost to Follow-Up**		**Remaining Participants**	
	**Control (N=52)**	**HD (N=85)**	**Control (N=12)**	**HD (N=35)**	**Control (N=40)**	**HD (N=51)**
**Age (years)**	50.65 (10.20)	52.06 (10.84)	49.74 (9.19)	51.77 (11.17)	50.92 (10.58)	52.24 (10.62)
[Range]	[26.15 - 69.96]	[23.19 - 78.06]	[37.06 - 66.58]	[25.71 - 78.06]	[26.15 - 69.96]	[23.19 - 68.98]
**Sex**	27F/25M	33F/52M	9F/3M	11F/24M	18F/22M	23F/28M
**Education**	14.06 (2.83)	13.98 (2.92)	14.00 (2.59)	14.06 (2.90)	14.07 (2.93)	13.94 (2.93)
[Range]	[8.00 - 24.00]	[9.00 - 20.00]	[8.00 - 18.00]	[10.00 - 19.00]	[11.00 - 24.00]	[9.00 - 20.00]

Supplementary Table 4: Missing data in classic cognitive tests

Variable	# Missing M0	% Missing M0	# Missing M1	% Missing M1	# Missing M1-M12	% Missing M1-M12
**Stroop Colour**	23	16.79	35	26.92	28	15.38
**Stroop Word**	23	16.79	35	26.92	28	15.38
**Stroop Interference**	23	16.79	35	26.92	28	15.38
**Letter Fluency**	34	24.82	45	34.62	38	20.88
**Categorical Fluency**	17	12.41	13	10.00	20	10.99
**SDMT**	1	0.73	3	2.31	3	1.65
**MDRS**	8	5.84	3	2.31	4	2.20
**HVLMT IR**	3	2.19	2	1.54	2	1.10
**HVLMT DR**	3	2.19	2	1.54	2	1.10
**cUHDRS**	23	16.79			22	12.09

MDRS Mattis Dementia Rating Scale. SDMT Symbol Digit Modalities Test. HVLMT Hopkins Verbal Learning Memory Test. IR Immediate Recall. DR Delayed Recall
number of observation

Supplementary Table 5: Study participants in cross-sectional VBM and TBSS analyses

	Baseline SelfCog (N=85)	Baseline T1 (N=51)	Baseline Diffusion (N=32)	p value
**Age**	52.06 (10.85)	51.76 (11.72)	53.96 (10.21)	0.64
[Range]	[23.19 - 78.06]	[23.19 - 71.27]	[29.81 - 71.27]	
**Sex**	33F/F2M	16F/35M	11F/21M	0.67
**Education**	13.98 (2.92)	14.65 (2.75)	14.03 (2.92)	0.40
[Range]	[9.00 - 20.00]	[9.00 - 20.00]	[9.00 - 20.00]	
**DBS**	400.03 (97.81)	404.91 (108.22)	416.92 (97.91)	0.73
[Range]	[129.62 - 672.50]	[134.16 - 672.50]	[134.16 - 600.91]	
**Study Site**	16CAR/42CRE/8MAN/19MUN	0CAR/34CRE/0MAN/17MUN	0CAR/32CRE/0MAN/0MUN	
**TFC**	10.52 (1.78)	10.90 (1.64)	10.97 (1.75)	0.31
[Range]	[7.00 - 13.00]	[7.00 - 13.00]	[7.00 - 13.00]	
**TMS**	29.68 (14.58)	29.57 (14.57)	29.47 (12.96)	0.997
[Range]	[1.00 - 60.00]	[1.00 - 58.00]	[1.00 - 56.00]	
**cUHDRS**	10.36 (2.94)	10.81 (2.79)	10.42 (2.89)	0.67
[Range]	[3.35 - 16.17]	[4.08 – 16.00]	[4.08 - 15.45]	

F Female. M Male. DBS Disease Burden Score. CAR Cardiff. CRE Creteil. MAN Manchester. MUN Munster. TFC Total Functional Capacity. TMS Total Motor Score. cUHDRS composite Unified Huntington Disease Rating Scale;
Disease Burden Score = Age x (CAG repeat – 35.5)

Supplementary Table 6: Demographics for HD patients in longitudinal SelfCog and MRI analysis
Unless otherwise specified. quantitative values are means ± standard deviations

	Longitudinal SelfCog (N=51)	Longitudinal T1 (N=24)	Longitudinal Diffusion (N=20)	p value
**Age**	53.29 (10.62)	54.75 (10.54)	53.58 (10.56)	0.86
[Range]	[24.23 - 69.99]	[30.97 - 69.99]	[30.97 - 68.46]	
**Sex**	23F/28M	8F/16M	8F/12M	0.62
**Education**	13.94 (2.93)	14.88 (3.00)	14.70 (3.11)	0.38
[Range]	[9.00 - 20.00]	[9.00 - 20.00]	[9.00 - 20.00]	
**DBS**	409.34 (109.99)	407.70 (116.01)	410.57 (116.38)	0.996
[Range]	[137.27 - 699.49]	[139.38 - 617.85]	[139.38 - 617.85]	
**Study_Site (CAR/CRE/MAN/MUN)**	10/31/5/5	0/24/0/0	0/20/0/0	
**TFC**	9.77 (2.29)	10.67 (2.20)	10.80 (1.99)	0.11
[Range]	[5.00 - 13.00]	[5.00 - 13.00]	[7.00 - 13.00]	
**TMS**	31.04 (16.45)	27.75 (14.46)	28.25 (13.02)	0.62
[Range]	[1.00 - 67.00]	[1.00 - 56.00]	[6.00 - 45.00]	
**cUHDRS**	9.73 (3.28)	10.35 (3.21)	10.36 (2.76)	0.64
[Range]	[2.78 - 16.16]	[2.82 - 16.16]	[5.77 - 15.15]	

F Female. M Male. DBS Disease Burden Score. CAR Cardiff. CRE Creteil. MAN Manchester. MUN Munster. TFC Total Functional Capacity. TMS Total Motor Score. cUHDRS composite Unified Huntington Disease Rating Scale; DBS Disease Burden Score = Age x (CAG repeat – 35.5)

Supplementary Table 7: Missing response (in percentage) obtained in the SelfCog battery in Huntington's Disease participants and Controls at Month 0 (M0), Month 1 (M1) and Month 12 (M12) sessions.

Status	Session	Mean	Standard deviation	min	max
Control	M0	0.54	0.71	0	3.5
Control	M1	0.32	0.50	0	2.5
Control	M12	0.54	1.37	0	8.5
HD	M0	4.38	5.97	0	26.5
HD	M1	3.10	4.44	0	24
HD	M12	4.10	5.63	0	25.5

Min minimum, max maximum

Supplementary Table 8: Clusters showing decreased grey matter integrity associated with cognitive scores in Huntington's disease participants at M0.

	MNI coordinates				
**GM clusters**	**x**	**y**	**z**	**cluster size**	**Corrected P**
		**LANGUAGE - ACCURACY**			
L lingual g	-26	-60	-8	5340	0.007
L putamen	-26	2	-2	2089	0.011
R putamen	24	10	-6	2061	0.001
R middle occipital lobe	28	-78	30	985	0.019
R inferior temporal lobe	48	-54	-12	675	0.029
R middle occipital lobe	28	-92	6	282	0.032
L middle temporal lobe	-52	-54	18	265	0.029
R hippocampus	24	-30	-6	38	0.046
R inferior temporal lobe	44	2	-34	24	0.045
R lingual g	12	-82	0	15	0.049
R inferior temporal lobe	56	-4	-28	9	0.049
R inferior temporal lobe	48	-12	-28	7	0.048
		**MEMORY - ACCURACY**			
L precentral g	-46	-6	44	5692	0.007
L precentral g	-26	-16	62	329	0.027
L inferior occipital lobe	-38	-84	-8	33	0.042
R middle frontal lobe	52	0	54	30	0.043
R precentral g	44	-8	54	1	0.05
		**MDRS**			
L inferior orbit frontal lobe	-26	28	-8	1426	0.008
R frontal WM	28	30	-6	1049	0.012
L superior occipital lobe	-18	-74	42	266	0.037
L precentral g	-36	-24	58	243	0.038
R cuneus lobe	18	-70	24	102	0.043
R inferior temporal lobe	48	-54	-10	93	0.025
R superior temporal lobe	46	-32	18	48	0.042
L inferior occipital lobe	-20	-90	-6	43	0.04
L frontal white matter	-20	-10	62	42	0.044
L precentral g	-34	-6	46	26	0.04
L occipital WM	-24	-76	12	13	0.046
R subcortical WM	28	-20	6	10	0.044
R superior parietal lobe	22	-62	50	8	0.048
		**STROOP - COLOR**			
L fusiform g	-24	-74	-8	1737	0.01
R cuneus lobe	22	-64	28	418	0.028

L supramarginal g	-56	-38	32	405	0.027
R inferior occipital lobe	32	-78	-8	285	0.024
R insula	38	10	2	277	0.028
L subcortical WM	-28	16	4	230	0.036
L subcortical WM	-38	14	-16	218	0.031
L occipito-temporal WM	-26	-44	18	32	0.042
R inferior temporal lobe	48	-50	-8	28	0.035
L parahippocampal g	-26	6	-28	20	0.046
R lingual g	22	-86	-4	18	0.047
**STROOP - INTERFERENCE**					
L occipito-temporal WM	-32	-60	6	1007	0.013
L middle occipital lobe	-36	-74	30	87	0.033
L inferior temporal lobe	-50	-62	-8	28	0.045
R parahippocampal g	20	-20	-16	20	0.041
L middle occipital lobe	-40	-72	36	1	0.05
**SDMT**					
L putamen	-24	18	-6	265	0.032
L lingual g	-26	-64	-6	214	0.027
L occipital WM	-30	-66	24	191	0.028
L precentral g	-44	-14	56	76	0.036
L precentral g	-32	-8	46	23	0.044
R inferior temporal lobe	48	-52	-10	19	0.03
L supramarginal g	-64	-32	40	18	0.044
L inferior parietal lobe	-56	-30	50	18	0.045
L inferior parietal lobe	-54	-24	38	6	0.049
**HVLMT - IR**					
L precentral g	-28	-14	70	1579	0.01
R precentral g	46	0	32	468	0.025
R precentral g	32	-14	60	347	0.02
R precentral g	-52	6	26	314	0.025
L supramarginal g	-50	-26	36	78	0.034
L inferior tri frontal lobe	-42	34	14	65	0.042
L inferior parietal lobe	-53	-32	48	41	0.043
L subcortical WM	-26	28	0	35	0.044
L inferior temporal lobe	-44	6	-36	28	0.046
L superior temporal lobe	-64	-10	8	7	0.047
L middle frontal lobe	-44	44	26	7	0.049
R parietal WM	32	-16	36	2	0.05

Cluster size: number of voxels, L left, R right, g gyrus, MDRS Mattis Dementia Rating Scale, SDMT Symbol Digit Modalities Test, HVLMT – IR Hopkins Verbal Learning Memory Test – Immediate Recall

Grey matter clusters significantly associated with cognitive scores ($P_{TFCE} < 0.05$). Coordinates indicate the location of the cluster peak in Montreal Neurological (MNI) convention.

Supplementary Table 9: Clusters showing decreased white matter integrity associated with cognitive scores in Huntington's disease participants at M0

	MNI coordinates				
**WM clusters - FSL-TBSS**	**x**	**y**	**z**	**cluster size**	**Corrected P**
		**MDRS**			
Corpus callosum	-10	-30	25	9528	0.013
		**Stroop - Color**			
Corpus callosum	4	-15	25	941	0.039
Corpus callosum	-27	-76	11	123	0.048
		**Stroop - Interference**			
Corpus callosum	-25	-75	14	763	0.037
		**SDMT**			
Corpus callosum	-15	-29	29	1244	0.031
L Optic radiations	-30	-72	10	130	0.045
L Internal capsule	-27	-59	16	126	0.048
		**HVLMT - RI**			
Corpus callosum	2	-23	24	968	0.035
		**HVLMT - RD**			
Corpus callosum	5	-21	24	4194	0.016
L cingulum	-23	-48	1	18	0.049

Cluster size: number of voxels, L left, R right, g gyrus, MDRS Mattis Dementia Rating Scale, SDMT Symbol Digit Modalities Test, HVLMT – IR Hopkins Verbal Learning Memory Test – Immediate Recall

White matter clusters significantly associated with cognitive scores ($P_{TFCE} < 0.05$). Coordinates indicate the location of the cluster peak in Montreal Neurological (MNI) convention.

Supplementary Table 10: Practice effect on the SelfCog IES. The table presents the p-values associated with the effect of Group, of Time and of the interaction between Group and Time and the correlation between the first and second evaluations in each group of participants with the associated p-value.

IES	Status	Month 0	Month 1	Group	Time	Group * Time	r	p-value
**Cognitive Global**	**Control**	1.67 (0.41)	1.58 (0.35)	1.12 e^{-10}	0.01	0.21	0.60	1.59 e^{-5}
	**HD**	3.46 (1.78)	3.09 (1.64)				0.58	7.37 e^{-8}
**Visual**	**Control**	0.86 (0.39)	0.82 (0.28)	4.04 e^{-4}	0.50	0.58	0.44	2.00 e^{-3}
	**HD**	1.92 (1.17)	2.07 (2.49)				0.34	3.00 e^{-3}
**Language**	**Control**	3.59 (1.04)	3.33 (0.91)	1.15 e^{-9}	3.00 e^{-3}	0.12	0.48	5.9 e^{-4}
	**HD**	7.52 (5.15)	6.25 (3.12)				0.49	1.06 e^{-5}
**Executive**	**Control**	0.78 (0.29)	0.75 (0.38)	3.4 e^{-7}	0.33	0.38	0.42	4.00 e^{-3}
	**HD**	1.51 (0.89)	1.59 (0.97)				0.60	2.06 e^{-8}
**Memory**	**Control**	1.46 (0.40)	1.44 (0.46)	2.14 e^{-9}	3.00 e^{-3}	0.07	0.35	0.02
	**HD**	2.90 (1.72)	2.45 (1.45)				0.51	3.84 e^{-6}

Supplementary Table 11: Results of model comparisons testing the interaction of time and group (Controls and HD patients) and post-hoc comparisons testing the M1-M12 change in performance in HD patients. Means (and standard deviations) are presented for each test at each time of evaluation

Scores	Interaction	Controls					HD				
	Group * Time	Month 1	Month 12	Estimate	SE	p value	Month 1	Month 12	Estimate	SE	p value
**Cognitive Global**	**3.0 e^{-3}**	1.56 (0.34)	1.57 (0.37)	2.42 e^{-3}	0.15	0.99	2.83 (1.08)	3.47 (1.73)	0.62	0.13	**1.00 e^{-4}**
**Visual IES**	0.12	0.81 (0.26)	0.76 (0.23)	0.06	0.31	0.84	1.66 (1.39)	2.26 (2.72)	-0.582	0.27	**0.04**
**Language IES**	0.08	3.26 (0.90)	3.44 (1.03)	-0.17	0.36	0.64	5.88 (2.36)	6.92 (4.04)	-1.03	0.32	**1.70 e-3**
**Executive IES**	**4.74 e^{-2}**	0.78 (0.40)	0.70 (0.28)	0.09	0.11	0.41	1.42 (0.75)	1.63 (0.82)	-0.19	0.09	**0.04**
**Memory IES**	**0.02**	1.38 (0.40)	1.36 (0.45)	0.03	0.23	0.89	2.37 (1.14)	3.07 (2.38)	-0.68	0.2	**1.30 e-3**
**MDRS**	**0.02**	142.02 (2.01)	141.95 (1.99)	-0.1	0.78	0.9	134.00 (7.84)	131.41 (7.38)	-2.62	0.69	**3.00 e^{-4}**
**SDMT**	0.18	54.65 (10.12)	54.00 (10.63)	-0.42	0.76	0.58	30.82 (9.95)	28.82 (10.22)	-1.77	0.67	**0.01**
**Stroop Colour**	**4.97 e^{-2}**	80.61 (11.66)	81.93 (11.17)	1.57	1.29	0.24	52.22 (13.63)	50.10 (12.33)	-1.86	1.15	0.11
**Stroop Word**	**0.02**	100.42 (12.32)	103.78 (14.80)	3.56	1.8	0.05	68.84 (17.48)	66.42 (14.89)	-2.21	1.59	0.17
**Stroop Interference**	**0.04**	47.91 (9.82)	48.32 (10.39)	0.7	1	0.49	29.14 (9.04)	26.76 (9.05)	-2.09	0.89	**0.02**
**Letter Fluency**	0.29	45.78 (9.87)	45.00 (11.15)	-0.88	1.24	0.48	30.68 (10.65)	28.15 (10.16)	-2.63	1.1	**0.02**
**Categorical Fluency**	0.09	23.08 (6.42)	23.58 (5.08)	0.55	0.76	0.47	14.69 (5.27)	13.44 (4.86)	-1.2	0.67	0.08
**HVLMT IR**	0.53	29.53 (3.36)	29.80 (3.88)	0.31	0.61	0.61	20.63 (4.48)	20.39 (5.48)	-0.2	0.54	0.71
**HVLMT DR**	0.24	10.75 (1.28)	11.10 (1.34)	0.34	0.35	0.33	6.78 (2.84)	6.58 (2.76)	-0.2	0.31	0.52

Supplementary Table 12: Results of model comparisons testing the main effect of time. interaction of time and group (Controls and HD patients) and post-hoc comparisons testing the M1-M12 change in performance in Controls and in Controls and in HD patients for accuracy and response time in the SelfCog battery. Means (and standard deviations) are presented for each test at each time of evaluation

Scores	Main Effects and Interaction			Controls					HD				
	**Group**	**Time**	**Group * Time**	**Month 1**	**Month 12**	**Estimate**	**SE**	**p value**	**Month 1**	**Month 12**	**Estimate**	**SE**	**p value**
**Mean Visual RT**	**$8.66\ e^{-7}$**	0.10	0.10	0.75 (0.25)	0.70 (0.22)	0.05	0.07	0.47	1.25 (0.56)	1.37 (0.57)	-0.11	0.07	0.10
**Mean Language RT**	**$4.72\ e^{-9}$**	0.08	0.55	2.69 (0.66)	2.80 (0.81)	-0.10	0.13	0.44	3.85 (1.07)	4.06 (1.11)	-0.20	0.11	0.08
**Mean Executive RT**	**$5.42\ e^{-8}$**	**0.02**	**0.02**	0.71 (0.25)	0.67 (0.23)	0.06	0.06	0.37	1.13 (0.45)	1.28 (0.45)	-0.14	0.06	**0.02**
**Mean Memory RT**	**$2.45\ e^{-5}$**	**0.01**	0.13	1.11 (0.26)	1.15 (0.35)	-0.02	0.07	0.77	1.60 (0.63)	1.78 (0.77)	-0.17	0.06	**0.01**
**Global RT**	**$1.01\ e^{-10}$**	**$4.69\ e^{-3}$**	0.06	1.32 (0.26)	1.33 (0.30)	$-1.87\ e^{-3}$	0.06	0.98	1.96 (0.55)	2.12 (0.60)	-0.15	0.05	**$4.70\ e^{-3}$**
**Mean Visual Accuracy**	$1.27\ e^{-3}$	0.26	0.40	0.92 (0.05)	0.92 (0.05)	$2.67\ e^{-3}$	0.02	0.90	0.82 (0.12)	0.80 (0.19)	-0.02	0.02	0.26
**Mean Language Accuracy**	$1.44\ e^{-6}$	**0.03**	0.34	0.83 (0.08)	0.82 (0.08)	-0.01	0.02	0.48	0.69 (0.15)	0.65 (0.16)	-0.04	0.02	**0.03**
**Mean Executive Accuracy**	$9.04\ e^{-4}$	0.16	0.28	0.96 (0.09)	0.96 (0.06)	$3.75\ e^{-3}$	0.02	0.84	0.86 (0.17)	0.83 (0.15)	-0.02	0.02	0.16
**Mean Memory Accuracy**	$2.62\ e^{-6}$	0.07	**0.01**	0.82 (0.09)	0.86 (0.09)	0.03	0.02	0.09	0.70 (0.12)	0.67 (0.15)	-0.03	0.02	0.07
**Global Accuracy**	**$3.89\ e^{-8}$**	**$2.71\ e^{-3}$**	**0.01**	0.88 (0.05)	0.89 (0.05)	$6.12\ e^{-3}$	0.01	0.55	0.77 (0.10)	0.74 (0.12)	-0.03	$9.00\ e^{-3}$	**$2.70\ e^{-3}$**
