# Supplementary material for: A new approach to digitized cognitive monitoring: validity of the SelfCog in Huntington’s disease
Source: Brain Commun. 2023 Mar 6;5(2):fcad043. doi: 10.1093/braincomms/fcad043 (PMC10018460; doi:10.1093/braincomms/fcad043)
Supplement: fcad043_Supplementary_Data [file fcad043_supplementary_data.zip › Supplementary Appendix 1 selfcog_description.pdf]

Appendix S1: SelfCog construction and procedure

1. Stimuli

Five hundred and eighty-five black line drawings were designed by a graphic designer, homogenised in their style and centred in a white square with a width and height of 600 pixels. Validation of the images was undertaken through a SurveyMonkey (https://fr.surveymonkey.com) in both French and English. The first step consisted of a selection of a set of imageable words (n=543) for use in the diverse cognitive tests. We hired a graphic designer artist, Marie Lévi, to draw these images. One-hundred and eighty French native-speakers (46 male; 106 females, mean age: 35 ±15.6 range: 18-75; mean education: 15 years ± 15 range: 9-20) were asked to name the pictures. The instruction was to "*give the first name that comes to mind when you look at the following object*". A cut-off (80% of correct recognition) was used. A second set of 86 new images were drawn and submitted to 84 new French native-speakers to replace the pictures that did not reached the 80% recognition threshold. The second group of participants was matched to the first one on age (mean age 42 years old±18, [20-79]), sex-ratio (27M/57F) and on educational level (mean age 14.8 years old±3.5, [9-20]). Altogether, this allowed the constitution of a bank of 555 correctly-named pictures. The design of the English version required translation and adaption to the English culture. To control the names of the pictures for frequency in the English language, number of syllables and the first syllable (important for the language assessment), 75 additional pictures were drawn and tested on 19 English native-speakers. Twenty-nine pictures were retained as they reached the cut-off 80% of correct recognition. In German, the whole pool of picture was correctly named by two pilot subjects which therefore did not trigger the creation of additional pictures.

2. SelfCog subtests presentation

1. ***Motor speed:*** Participants are instructed to press the key on the side on which the image appears as soon as they see it. A score is given for errors and response times for left and right responses, allowing the detection of laterality differences. The motor response time score serves as a response time baseline to be subtracted from the cognitive tasks, as it provides a measure of the amount of time required to process and react to a simple stimulus.
2. ***Visuospatial***: Participants are instructed to determine whether the two images correspond to the same object (left for "no", right for "yes"). This subtest evaluates different types of knowledge that may lead to impairments in visual tasks. It comprises conceptual and visual abilities with trials including different exemplars of the same object, mental rotation with the same object presented in different positions, semantically unrelated but visually similar, and semantically related but visually dissimilar objects.
3. ***Language***: Participants must indicate whether the two objects start with the same syllable by pressing either left for "no" or right for "yes". This test targets both language perception and production, requiring matching the result of the two processes.
4. ***Executive Function***: Participants are instructed to press the key on the side where the image of an animal appears (task 1). In half of the trials, both drawings correspond to animals or objects (task 2). In this condition, participants must simply press the right key. This subtest requires managing two tasks (tasks 1 and 2). Scores can be calculated for task switching trials and non-task switching trials. Impairment in executive function should lead to greater switch costs.
5. ***Memory***: The participant is asked to press the key on the side on which the picture was previously seen. At each trial, two pictures are presented, one which has previously been shown only once and one which has never been seen. Episodic short-term memory is measured with the presentation of the same picture presented within the same test in few

trials previously. Delayed memory is evaluated by the presentation of pictures seen in the previous subtests. Each of these conditions consists of half of the subtest.

3. Pilot studies in French cohorts

The first version of the SelfCog consisted of 400 trials (80 trials per subtest). 30 healthy volunteers, reporting no neurological or psychiatric disorders, and 30 neurologic participants (referred to the neuropsychology unit at the Henri Mondor hospital neurology department: stroke, Huntington's Disease participants) participated in the study to validate the test material. All participants performed all the entire SelfCog battery and a battery of classical paper-and-pencil tests. However, the SelfCog's evaluation lasted more than 30 minutes and up to an hour in participants with neurological condition. Global cognition scores assessed by the SelfCog were significantly correlated to the MMSE and Mattis Dementia Rating Scales in favour to convergent validity. Correlations between specific cognitive functions of the SelfCog and appropriate battery tests were significant. Internal consistency was found to be satisfactory for all subtests with a Cronbach's alpha superior to 0.95 (motor: 0.98; visual: 0.97; executive: 0.96; language: 0.97; memory: 0.95).

Following the results of the pilot study, we modify the SelfCog battery:

- To shortcut the duration of the assessment, a time lock was introduced for each subtest. Based on response times obtained in the pilot study in neurologic patients, we defined a limit of response time for each subtest. The display of each item on the screen of the computer should not exceed the answer time of more than 90% responses by condition.
- To shortcut the duration of the assessment, we also decided to decrease the number of trial per subtest. Based on data from the pilot study, a power analysis (t-test) was conducted and revealed that similar results could be obtained with 40 trials.

- The new version allowed measurement of missing responses (when participant do not response in a defined time) based on the implementation of a time-lock.
