# Supplementary material for: A new approach to digitized cognitive monitoring: validity of the SelfCog in Huntington’s disease
Source: Brain Commun. 2023 Mar 6;5(2):fcad043. doi: 10.1093/braincomms/fcad043 (PMC10018460; doi:10.1093/braincomms/fcad043)
Supplement: fcad043_Supplementary_Data [file fcad043_supplementary_data.zip › Supplementary Appendix 2 MRI data acquisition.pdf]

Appendix S2: MRI data acquisition, processing and analysis

Brain MRI acquisition was performed at two centres. At Henri Mondor Hospital (Créteil, France), participants underwent a high-resolution brain MRI scan on a Siemens Skyra including T1 3D anatomical MP-RAGE images (repetition time: 2300 ms; echo time: 2900 ms; inversion time: 900 ms; flip angle: 9°; acquisition matrix: 256 x 240; slice thickness: 1.2 mm, no inter-slice gap, 176 sagittal sections). In addition, a diffusion tensor sequence was also performed using echo-planar imaging (repetition time: 8900 ms, echo time: 75 ms; acquisition matrix: 116 x 116; percent phase field of view = 100; slice thickness = 2.5 mm; voxel resolution = 2.5, 2.5, 2.5 mm^3). Sixty-four diffusion images weighted with a b-value of 1000 sec mm^{-2} and a volume with no diffusion gradient was acquired. At the George Huntington Institute (Muenster, Germany), participants underwent a high-resolution brain MRI scan on a Philips Medical Systems including T1 3D anatomical MP-RAGE images (repetition time: 6770 ms; echo time: 3130 ms; inversion time: 900 ms; flip angle: 9°; acquisition matrix: 256 x 256; slice thickness: 1.2 mm, inter-slice gap: 1.2 mm, 170 sagittal sections).

Structural 3D-T1-weighted data preprocessing

At baseline, structural data were analyzed with the standard processing stream of FSL-VBM (1–3) (https://fsl.fmrib.ox.ac.uk/fsl/fslwiki/FSLVBM). First, structural images were brain extracted and grey matter segmented before being registered to the 2-mm MNI 152 standard space using nonlinear registration. The resulting images were averaged and flipped along the x-axis to create a left-right symmetric, study-specific grey matter template. Second, all native grey matter images were nonlinearly registered to this study-specific template and 'modulated' to correct for local expansion or contraction due to the nonlinear component of the spatial transformation. The modulated grey matter images were then smoothed with an isotropic Gaussian kernel with a sigma of 3 mm.

We employed for the longitudinal analysis a simple modification to the FSL-VBM processing stream in which we first registered baseline and follow-up images in their halfway space and using a study-specific template (3,4). Each patient's structural images were brain-extracted (5), linearly registered to the structural image from the second follow-up visit and averaged to create a subject specific template in a common space. All original structural images were then linearly registered to this template. These steps were done by SIENA and allow to obtain the percentage of brain atrophy over one-year per patient. The average images were then brain-extracted using BET. Next, tissue-type segmentation was carried out on the subject mean image using FAST (6). The resulting gray-matter partial volume images were then aligned to MNI-152 standard space using affine registration. The resulting images were averaged to create a study-specific template. The brain extraction and segmentation steps were then repeated on the rigidly aligned structural scans from each scanning session. The segmented native grey images were then non-linearly registered to the template using the transformations calculated from the averaged images. The segmented images were then smoothed with an isotropic Gaussian kernel with a sigma of 3 mm. We then subtracted the baseline from the follow-up images to obtain a difference image for each subject.

Diffusion tensor imaging (DTI) preprocessing

Each HD patient's DTI scans were corrected for head motions and eddy-currents and then brain-extracted. Fractional anisotropy (FA) maps were created using DTIFIT by applying a diffusion tensor model to each voxel (7). We used a whole-brain voxel wise analysis of the FA data carried out with TBSS (8), as included in the FSL software package (2); http://fsl.fmrib.ox.ac.uk/fsl/fslwiki/FSL). At baseline, all individual FA maps were aligned to an averaged FA template (FMRI58_FA template) in standard space using a non-linear registration. Correct registration was visual checked for each patient. Next, an average FA map

was created and a skeleton map representing the centre of the white matter (FA > 0.2) common to all patients computed. Finally, patient's registered FA maps were projected into the skeleton.

For the longitudinal analysis, baseline and follow-up FA images were registered in a study-specific template space, whereby the baseline and follow-up FA images were first registered into their halfway space for each HD patients. The average FA image between baseline and follow-up in halfway-space was then non-linearly registered onto the FMRIB-FA template. Both wraps were concatenated and applied to create a study-specific template. Finally, baseline and follow-up FA images for each subject were non-linearly registered to this study-specific template. To estimate the WM microstructural change over one year for each participant, we subtracted each projected skeletonized FA image at follow-up from their respective baseline image. The difference images (obtained from images subtraction) were then used in our statistical models.

References

1. Good CD, Johnsrude IS, Ashburner J, Henson RN, Friston KJ, Frackowiak RS. A voxel-based morphometric study of ageing in 465 normal adult human brains. Neuroimage. 2001 Jul;14(1 Pt 1):21–36.

2. Smith SM, Jenkinson M, Woolrich MW, Beckmann CF, Behrens TEJ, Johansen-Berg H, et al. Advances in functional and structural MR image analysis and implementation as FSL. NeuroImage. 2004;23, Supplement 1:S208–19.

3. Douaud G, Smith S, Jenkinson M, Behrens T, Johansen-Berg H, Vickers J, et al. Anatomically related grey and white matter abnormalities in adolescent-onset schizophrenia. Brain. 2007 Sep;130(Pt 9):2375–86.

4. Thomas AG, Marrett S, Saad ZS, Ruff DA, Martin A, Bandettini PA. Functional but not structural changes associated with learning: an exploration of longitudinal voxel-based morphometry (VBM). Neuroimage. 2009 Oct 15;48(1):117–25.

5. Fast robust automated brain extraction - Smith - 2002 - Human Brain Mapping - Wiley Online Library [Internet]. [cited 2021 Oct 27]. Available from: https://onlinelibrary.wiley.com/doi/full/10.1002/hbm.10062

6. Zhang Y, Brady M, Smith S. Segmentation of brain MR images through a hidden Markov random field model and the expectation-maximization algorithm. IEEE Trans Med Imaging. 2001 Jan;20(1):45–57.

7. Behrens TEJ, Woolrich MW, Jenkinson M, Johansen-Berg H, Nunes RG, Clare S, et al. Characterization and propagation of uncertainty in diffusion-weighted MR imaging. Magn Reson Med. 2003 Nov;50(5):1077–88.

8. Smith SM, Jenkinson M, Johansen-Berg H, Rueckert D, Nichols TE, Mackay CE, et al. Tract-based spatial statistics: voxelwise analysis of multi-subject diffusion data. Neuroimage. 2006 Jul 15;31(4):1487–505.
